# Recent Developments in the Optimization of the Bulk Heterojunction Morphology of Polymer: Fullerene Solar Cells

**DOI:** 10.3390/ma11122560

**Published:** 2018-12-16

**Authors:** Hugo Gaspar, Flávio Figueira, Luiz Pereira, Adélio Mendes, Júlio C. Viana, Gabriel Bernardo

**Affiliations:** 1 IPC/i3N—Institute for Polymers and Composites, University of Minho, Campus de Azurém, 4800-058 Guimarães, Portugal; hugo.da.silva.gaspar@gmail.com (H.G.); jcv@dep.uminho.pt (J.C.V.); 2 QOPNA, Departament of Chemistry, University of Aveiro, 3810-193 Aveiro, Portugal; ffigueira@ua.pt; 3 Department of Physics and i3N—Institute for Nanostructures, Nanomodelling and Nanofabrication, University of Aveiro, 3810-193 Aveiro, Portugal; luiz@ua.pt; 4 LEPABE, Department of Chemical Engineering, University of Porto, 4200-465 Porto, Portugal; mendes@fe.up.pt

**Keywords:** organic photovoltaics, fullerenes

## Abstract

Organic photovoltaic (OPV) devices, made with semiconducting polymers, have recently attained a power conversion efficiency (PCE) over 14% in single junction cells and over 17% in tandem cells. These high performances, together with the suitability of the technology to inexpensive large-scale manufacture, over lightweight and flexible plastic substrates using roll-to-roll (R2R) processing, place the technology amongst the most promising for future harvesting of solar energy. Although OPVs using non-fullerene acceptors have recently outperformed their fullerene-based counterparts, the research in the development of new fullerenes and in the improvement of the bulk-heterojunction (BHJ) morphology and device efficiency of polymer:fullerene solar cells remains very active. In this review article, the most relevant research works performed over the last 3 years, that is, since the year 2016 onwards, in the field of fullerene-based polymer solar cells based on the copolymers PTB7, PTB7-Th (also known as PBDTTT-EFT) and PffBT4T-2OD, are presented and discussed. This review is primarily focused on studies that involve the improvement of the BHJ morphology, efficiency and stability of small active area devices (typically < 15 mm^2^), through the use of different processing strategies such as the use of different fullerene acceptors, different processing solvents and additives and different thermal treatments.

## 1. Introduction

Organic photovoltaics (OPVs) represent a promising approach to generate renewable energy. Compared with currently available technologies, OPVs can be easily manufactured over much larger areas, on lightweight plastic substrates with high flexibility, using high-throughput roll-to-roll (R2R) compatible processing technologies [1,2,3,4,5,6]. These capabilities strongly suggest that OPVs will enable large reductions in module fabrication cost and a consequent reduction on the energy payback time. The low production costs associated with OPVs might be the key for opening solar energy to new markets such as, for example, in rural communities and developing countries with poor transmission infrastructures by allowing people to invest and generate their own electricity. On the other hand, gadget market, in a low power, low cost energy conversion and wearable structure incorporation, are perhaps the most suitable application.

Although the progress in OPVs has been slower than for example in perovskite solar cells, the efficiency of single junction polymer solar cells has been increasing steadily in the last 10–15 years, evolving from 5% in 2005 to above 14% in 2017 [7,8] and tandem cells have reached an even higher efficiency, above 17% [9]. Criteria such as efficiency, lifetime and cost, need to be satisfied to successfully commercialize large scale organic photovoltaics but considering the steady progress that has been happening in the field, the future of the technology looks bright.

Polymer solar cells were dominated, for over two decades, by donor: acceptor blends based on fullerene acceptors. However, due to rapid developments in non-fullerene (NF) small molecule acceptors, in recent years NF acceptors have outperformed the fullerene acceptors and a large number of reviews have recently addressed the application of this newer type of acceptors in OPVs [10,11,12,13,14,15]. Despite this, the research activity in the field of fullerene-based polymer solar cells remains very intense and important developments have occurred in the last few years.

This review focus mainly on the efforts performed over the last three years to improve the bulk-heterojunction (BHJ) morphology and the efficiency of small active area (typically < 15 mm^2^) polymer:fullerene solar cells based on three of the most recent and highest efficiency low bandgap polymers, namely PTB7, PTB7-Th (also known as PBDTTT-EFT) and PffBT4T-2OD. These studies include either changing the morphology through the use of different processing strategies (different solvents and additives; different thermal treatments; different donor: acceptor ratios; etc.) or changing the morphology using other fullerenes different from the standards PC_71_BM ([6,6]-phenyl-C_71_-butyric acid methyl ester) and PC_61_BM ([6,6]-phenyl-C_61_-butyric acid methyl ester). Strategies to improve the morphological stability of the BHJ are also reported. As a benchmark for high performance, when considering studies involving the standard fullerenes PC_71_BM and PC_61_BM, mostly research works reporting power conversion efficiencies (PCE) of 8% or higher are reviewed. This requirement is relaxed when considering studies involving other fullerenes.

This review provides first a general background to the field (Chapter 1) while Chapter 2 goes deeper on the polymers and fullerenes considered along the review; Chapter 3 discusses the general use of additives in OPVs and Chapters 4–6 discuss the main works based on respectively polymers PTB7, PTB7-Th and PffBT4T-2OD; finally, Chapter 7 draws the main conclusions and future perspectives.

### 1.1. Device Architectures

**Figure 1:**
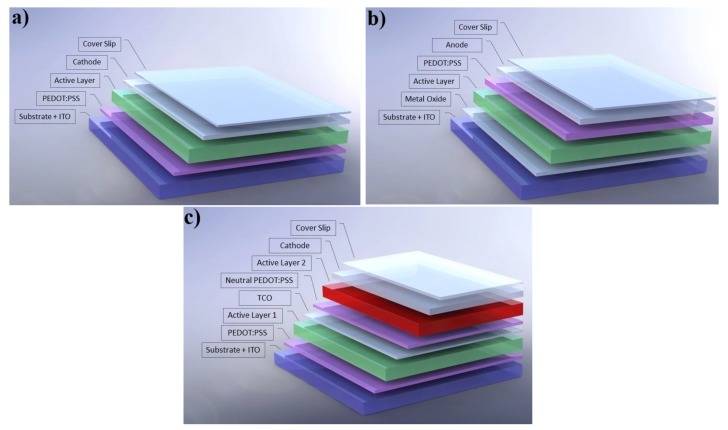
Architectures used in the construction of OPV devices: (**a**) Standard (Normal); (**b**) Inverted and (**c**) Tandem. TCO and ITO stand respectively for “Transparent Conductive Oxide” and “Indium Tin Oxide.”

OPV devices can be typically manufactured with three different architectures, as sketched in Figure 1: standard (normal); inverted and tandem.

The device architecture of a normal or standard device is shown in Figure 1a. In this configuration, ITO is usually coated with a hole transport layer/electron blocking layer (HTL/EBL) of poly(3,4-ethylenedioxythiophene)-poly(styrenesulfonate) (PEDOT:PSS). The BHJ layer is coated on top of the HTL and finally a low work function metal electrode (usually, calcium and aluminium) is deposited on top for collecting the electrons generated in the BHJ (cathode). Suitable improvement in the cathode can also be done by using dielectric materials like LiF that promote a remarkable decrease in the electrode work function by tunnelling process. The exposure of the acidic PEDOT:PSS to ITO has been reported as a potential stability problem, especially at high temperatures and high relative humidities.

Although the standard geometry is still the most widely used, its limited operational lifetime is a disadvantage. Compared to the standard device, the inverted OPV configuration shown in Figure 1b has some associated advantages. In this configuration, the ITO acts as an electron collector (cathode) and is usually coated with a transparent metal oxide layer like zinc oxide or titanium dioxide. The active BHJ layer is deposited on top of the metal oxide layer, followed by a HTL (usually PEDOT:PSS) and the device is finalized with the deposition of a high work-function metal anode. Compared to the normal structure, the inverted structure has the advantage of allowing the use, as top anode, of an ambient stable high work-function metal such as gold (Au), silver (Ag) and copper (Cu), which can be deposited under normal ambient atmosphere conditions using scalable deposition technologies such as inkjet printing [16,17,18]. A recent and extensive review on this particular OPV device architecture can be found elsewhere [19].

The device architecture of a tandem device is represented in Figure 1c. The tandem architecture integrates two or more sub-cells, integrating photoactive layers with complementary absorption spectra, stacked in series. The main advantage of the tandem architecture, compared to the single-junction architecture, is that it can maximize the proportion of the solar irradiation that is absorbed and used to generate electricity. A recent study suggests that OPVs with PCE > 17% can be obtained using highly optimized tandem architectures [9].

### 1.2. Macroscopic Device Physics

**Figure 2:**
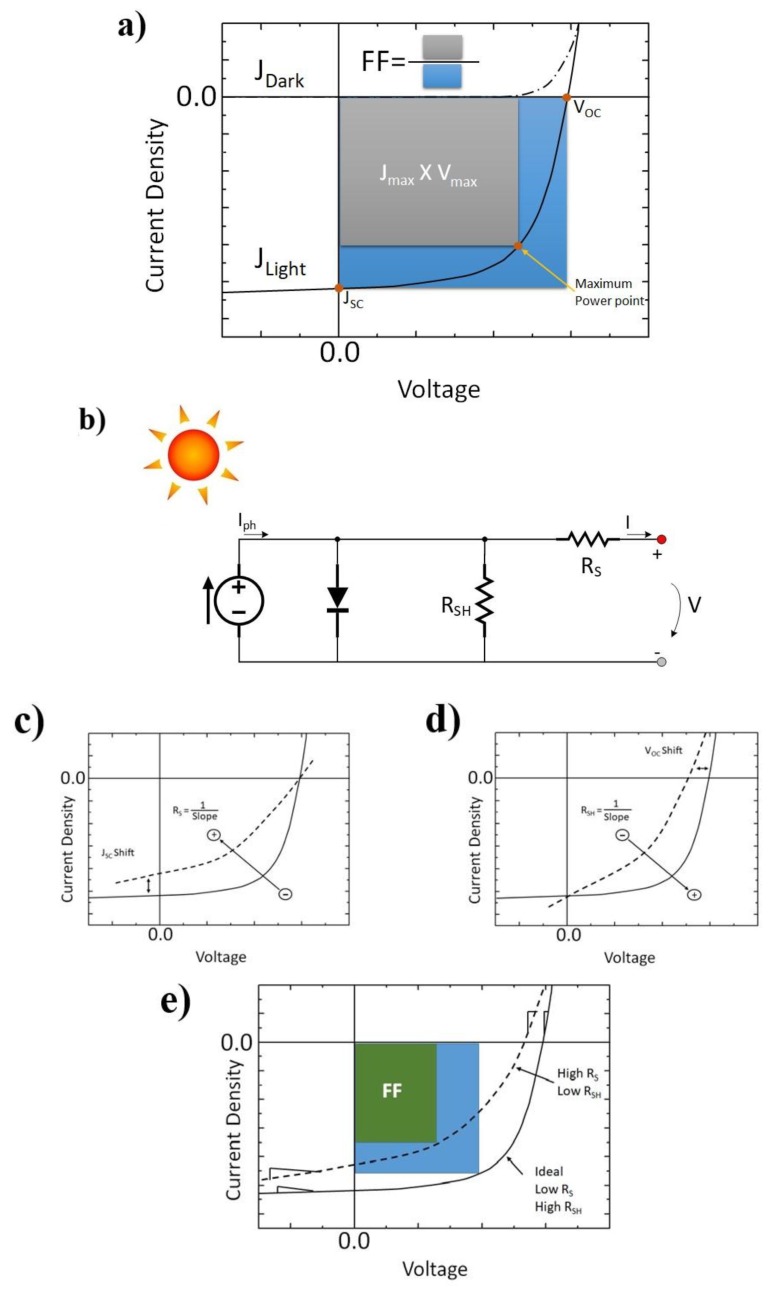
(**a**) Fundamental points in a solar cell I-V curve required for a full understanding of its figures of merit; (**b**) The usual OPV equivalent electrical circuit; (**c**) Impact of the variation of the series resistance (*R_s_*) on *FF*; (**d**) Impact of the variation of the shunt resistance (*R_sh_*) on the *FF*; (**e**) real I-V curve (as usually found) where both *R_s_* and *R_sh_* are not ideal.

Figure 2a shows the typical current density-potential (J-V) curve for an OPV device under dark and illumination conditions. The power conversion efficiency (*PCE*) of the device is the product of three figures of merit, namely short-circuit current (*J_sc_*), open-circuit voltage (*V_oc_*) and fill factor (*FF*) divided by the incident power radiation *P_i_*, that is: *PCE = (J_sc_* × *V_oc_* × *FF)/P_i_*.

Improving the figures of merit of an OPV requires a critical analysis of its macroscopic behaviour. Figure 2b shows the equivalent circuit for a solar cell. In this, *J_ph_* is the photoinduced current density (all generated photocurrent from photon to electron energy transfer); *R_s_* is the series resistance; *R_sh_* is the shunt resistance; *J* is the current flow in the external load and *V* is the applied voltage.

The series resistance *R_s_* originates from the bulk resistance of the active layer, the bulk resistance of the electrodes and the contact resistances of all interfaces in the device. The value of *R_s_* determines where the current mainly flows (considering the potential range in the 4th quadrant): to the diode if *R_s_* is high or to the external load if *R_s_* is low. Therefore, in the fabrication of solar cells, ideally *R_s_* should be as low as possible (*R_s_* → 0), as an effective way to improve the performance of the device.

The shunt resistance *R_sh_* may originate from manufacturing or natural intrinsic defects and includes current leakage from pinholes in the film, current leakage from the edge of the cell, current leakage by energy levels acting as traps or electron-hole recombination. It has the effect of dividing the current in the equivalent circuit and therefore ideally the value of *R_sh_* should approach infinity so that there is no current loss in the device, that is, there is no current flow through *R_sh_*.

Figure 2c,d represents the impact of the series resistance and shunt resistance on the fill factor, considering all the remaining parameters as constant. The *FF* represents how “rectangular” the *J-V* curve is and it characterizes how “difficult” or how “easy” the photogenerated carriers can be extracted from the solar cell. The ideal value for *FF* is 100% when the *J-V* curve is a perfect rectangle—however in practice this never happens and in OPVs the *FF* values are typically in the range 40–80%. From Figure 2c it can be seen that at negative and low positive voltages the *J-V* curve is a straight line with slope 1/*R_sh_*; at intermediate positive voltages the *J-V* curve is an exponential line that is determined by the diode; and at higher positive voltages the *J-V* curve is another straight line with slope 1/*R_s_*. Therefore, to improve the *FF* value the shunt resistance should be as high as possible and the series resistance should be as low as possible. It must be noted that in a real device *R_s_* is higher than the ideal and *R_sh_* is lower than the ideal. Therefore, an I-V curve as shown in Figure 2e is the usual.

### 1.3. Relationship between Nanoscopic and Macroscopic Device Physics

**Figure 3:**
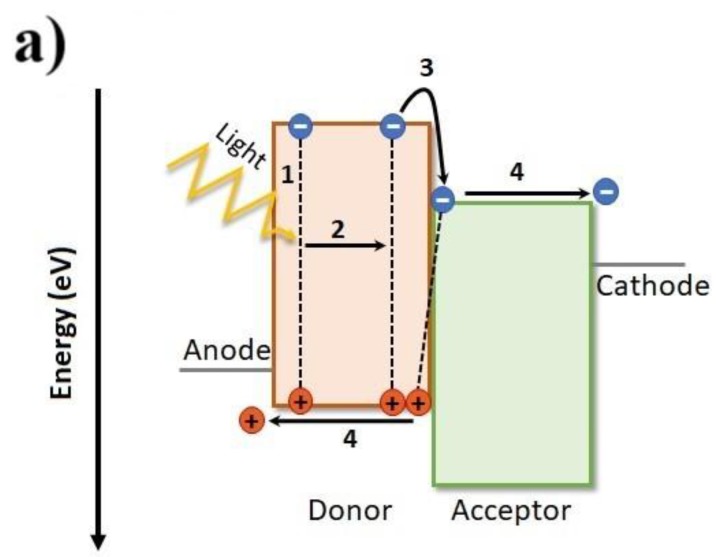

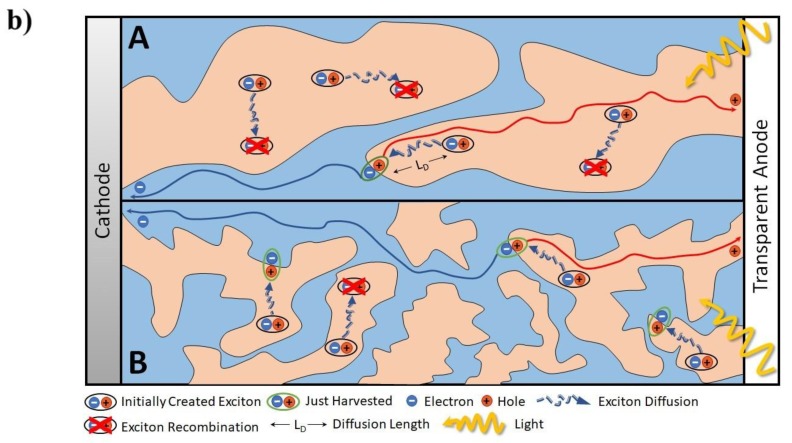
(**a**) Typical band diagram for a donor:acceptor BHJ in organic solar cells; (**b**) Photocurrent generation processes in two BHJs with very different morphologies: a bad morphology (A) and a good morphology (B).

The typical active layer in a OPV device is composed of a blend of an electron donating polymer and an electron acceptor which can be either fullerene-based (PC_71_BM, PC_61_BM or others) or a non-fullerene small organic molecule. This donor: acceptor blend is known as bulk-hetero-junction (BHJ). Figure 3a represents the typical band diagram for a donor: acceptor bulk-hetero-junction in an OPV device. Incident photons with energy higher than the bandgap (E_g_), that is, E > E_g_, are absorbed by the BHJ causing the generation of excitons, that is, electron-hole pairs (step 1 in Figure 3a). Typical binding energy values for excitons are on the order of 0.5 eV or larger [20,21]. Then, the excitons created must diffuse to a donor: acceptor interface where they dissociate efficiently into holes and electrons (steps 2 and 3 in Figure 3a). At this point, it is worth emphasizing that until a few years ago the LUMO-LUMO offset (ΔLUMO) between the donor and the acceptor was considered to be the driving force needed to split the tightly bound excitons and a ΔLUMO ≥ 0.3 V was regarded as the minimum necessary to ensure efficient charge transfer [22]. However, recent studies have shown that other factors also play their role and highly efficient OPVs can have ΔLUMO < 0.1 eV [23]. Finally, the separated electrons and holes must travel along the interpenetrating network towards the metal cathode and anode, respectively, where they are collected giving rise to a photocurrent and photo-voltage (step 4 in Figure 3a). Due to the low dielectric constant of conjugated polymers, the typical exciton diffusion length (*L_D_*) in a BHJ, that is, the average distance an exciton can diffuse through the material before recombination happens, is as low as ca. 10 nm [24,25,26,27,28], a length-scale that necessarily defines the optimal size of phase-separation for maximizing device efficiency.

This is illustrated in Figure 3b where the processes occurring in two very different BHJ morphologies are represented. In the ideal morphology, most of the excitons created can find nearby (distance < *L_D_*) a donor:acceptor interface where they dissociate generating free charge carriers and therefore the corresponding device has a high PCE. By contrast, in the morphology with very large phase segregation most of the excitons when created are too far away (distance > *L_D_*) from the nearest donor:acceptor interface and they recombine. Therefore, the ideal morphology for a BHJ is typically considered to be a bi-continuous and interpenetrating comb-like network morphology composed of donor-rich and acceptor-rich phases, with the donor phase smallest dimension of ~20 nm. Electrical carrier mobility depends on those domains. As widely accepted, the better figures of merit result from a compromise between D:A interface total area and D:A domains bulk volume. It is worth mentioning, at this point, that different experimental techniques can be used to probe the nano-morphology of BHJs. These include: Atomic Force Microscopy [29], High Resolution Electron Microscopy [30], Near-Edge X-ray Absorption Fine Structure (NEXAFS) spectroscopy [31], Grazing Incidence Wide and Small Angle X-ray Scattering (GI-WAXS/GI-SAXS) [32], Resonant Soft X-Ray Scattering (RSoXS) [33], Spectroscopic Ellipsometry [34,35], Neutron Reflectivity [36,37] and Small Angle Neutron Scattering (SANS) [38,39,40]; the discussion of these techniques is, however, beyond the scope of this review.

The open circuit voltage (*V_oc_*) [41,42,43,44] is primarily dependent on the energy difference between the LUMO level of the acceptor and the HOMO level of the donor: the higher the difference, the higher the *V_oc_*. Therefore, the main strategy usually followed to increase *V_oc_* consists in utilizing either fullerene acceptors with higher LUMO levels or donor polymers with lower HOMO levels. As long as the LUMO of the fullerene acceptor remains lower than the LUMO of the polymer donor by an amount sufficient to promote charge separation, raising the fullerene LUMO level or lowering the polymer HOMO level should increase the *V_oc_* and thus the PCE. The *V_oc_* is prone to various loss mechanisms that affect the device efficiency and these have recently received special attention [21,45,46,47].

The short-circuit current (*J_sc_*) [48,49,50,51] depends on several factors such as the amount of light absorbed, the efficiency of exciton and free charge generation, the charge-carrier mobility and the efficiency of charge extraction at the electrodes. The higher these variables are, the higher is the corresponding *J_sc_* value.

The fill factor (*FF*) [52,53,54,55], as mentioned above, depends on the series resistance, shunt resistance and diode. However, these also depend on a large number of factors that interact with each other intricately and for this reason the *FF* is more complex and less understood than the *V_oc_* and *J_sc_* [47,56,57,58]. Previous studies have shown that variables such as the blend composition, the blend morphology, the regioregularity of the conjugated polymer and the thickness of the BHJ influence significantly the *FF* affecting *R_s_* and *R_sh_* [52]. When the crystallinity of the blend layer increases, either by thermal annealing or through the use of a regioregular polymer, *R_s_* decreases [52,55]. When the thickness of the blend layer increases, *R_s_* increases and *R_sh_* decreases [52,59]. The quality of the two interfaces between the BHJ and the electrodes was also shown to play an important role in determining *FF* [52,60]. In practice, *FF* depends on the I-V shape in the 4th quadrant. As the D:A junction degrades (by molecular conformation and orientation and/or energetically inappropriate levels of D:A materials) the I-V behaviour becomes more space charge limited (SCLC) dependent and the I-V curve displays a typical “s-shape.” Common strategies to reduce *R_s_* and increase *R_sh_* include using buffer layers to reduce recombination [55], increasing the crystallinity of the BHJ materials [55,61], changing the donor: acceptor mass ratios [62] and optimizing the size of phase domains in the BHJ [55,61].

Among the several physical processes involved in the electrical power generation, the radiative (photons) absorption and charge collection at electrodes (involving the charge separation at D:A interfaces and electrical carriers transport in OPV volume) are often the most interesting for device improvement. In a simple model, the *J_sc_* value should be close to the *J_Ph,_* but from the equivalent electrical circuit it is easy to understand that *J_sc_* decreases, in a primary way, by the effect of series resistance *R_s_*. But, the I-V shape depends primarily on the *R_sh_*. In fact, this resistance comprehends all physical effects responsible for the electrical carrier recombination from the exciton separation (charge state at interface domain) until the collection at electrodes (from the electrical charge transport in volume). The value of *R_sh_* should, therefore, be as high as possible (as experimentally observed > 10^6^ Ω). On other hand, increasing the photogenerated carriers should increase the *J_sc_* value. We must therefore primarily search for donors (polymers) with reduced (as possible) bandgap (HOMO—LUMO difference) to increase the solar light absorption in the visible spectrum.

Donor-Acceptor (D:A) interface is also a key factor in the OPV energy performance. As soon as the bonded excitons reach the D:A interface, an extremely competitive process between separation (charge transfer—CT—states) and recombination will take place, determining how much efficient will be the free (net) charge creation. Once again, this process is almost (besides energy levels) dominated by the molecular structure/conformation of the donor/acceptor materials in the active film.

Regarding the bulk transport, a competitive framework between electrical carriers drift and recombination must occur. To allow the highest charge collection possible at electrodes, the drift time should be smaller as possible to a positive competition with the high probable electron-hole recombination due to the complex distribution of a high density of electrical active energy levels in the BHJ [63,64]. As demonstrated from transient photoconductivity (see for example, [65]), the characteristic drift time is proportional to $\frac{d^{3}}{2\mu E}$, where *d* is the organic layer thickness, *μ* the electrical carrier density and $E_{i}=\left(V_{OC}-V_{ext}\right)\times d^{-1}$ the internal electrical field, dependent on the *V_oc_* and the external applied voltage (*V_ext_*). An important aspect, is that the drift time is inversely proportional to the electrical carrier mobility. Increasing the degree of structural ordering of the D–A matrix, an increase of electrical charge mobility is expected (hopping process facilitated) with a consequent decrease of the drift time. This must allow the electrical carriers to drift towards electrodes before they can recombine, increasing the *FF*. Morphological ordering of the active layer is therefore desired.

All the referred parameters are intrinsically related to the donor and acceptor compounds used and their interaction in the solid state. Optimization and incrementation of these parameters require a deep comprehension of the device operation and photocurrent generation (J_PH_) as well as its limitations [66].

## 2. Polymers and Fullerenes

In polymer:fullerene solar cells the role of light absorption has traditionally been assigned to the polymer donor, for the simple reason that fullerenes do not absorb strongly in the visible and near-infrared region of the spectrum. For this reason, a large amount of research in the OPV field has focused on synthesizing new polymers with a small optical bandgap *E_g_* [67,68,69]. Besides the low optical bandgaps to broaden the absorption range into the infrared spectrum, polymers used as electron donors in polymer:fullerene solar cells should exhibit suitable LUMO energy levels for efficient electron transfer to the fullerene moieties and crystalline characteristics to ensure good charge mobility [68]. Three of the most recent and successful small band gap copolymers that, when compared with the “old reference” poly(3-hexylthiophene-2,5-diyl) (P3HT), *E_g_* ca. 2.1 eV [70], have a smaller energy gap (allowing more of the sun’s spectral emission to be harvested) and an higher ionization potential (leading to an increased *V_oc_* and thus greater PCE) are: (i) poly[[4,8-bis[(2-ethylhexyl)oxy] benzo[1,2-b:4,5-b’]dithiophene-2,6-diyl][3-fluoro-2-[(2-ethylhexyl)carbonyl]thieno [3–b]thiophenedi-yl]], commonly known as PTB7 (*E_g_* ca. 1.6 eV); (ii) poly[4,8-bis(5-(2-ethyl hexyl)thiophen-2-yl)benzo[1–b;4,5-b’]dithiophene-2,6-diyl-alt-(4-(2-ethylhexyl)-3-fluorothieno[3–b]thiophene-)-2-carboxylate-2–6-diyl)], commonly known as PTB7-Th or PBDTTT-EFT (*E_g_* ca. 1.6 eV) and iii) ((poly[(5,6-difluoro-2,1,3-benzothiadiazol-4,7-diyl)-alt-(3,3‴di(2-octyldodecyl) 2,2′;5′,2″;5″,2‴ -quaterthiophen-5,5‴-diyl), commonly known as PffBT4T-2OD (Eg = 1.65 eV). The *E_g_* values indicated are only approximate as they depend on the polymer molecular weight [71].

**Figure 4:**
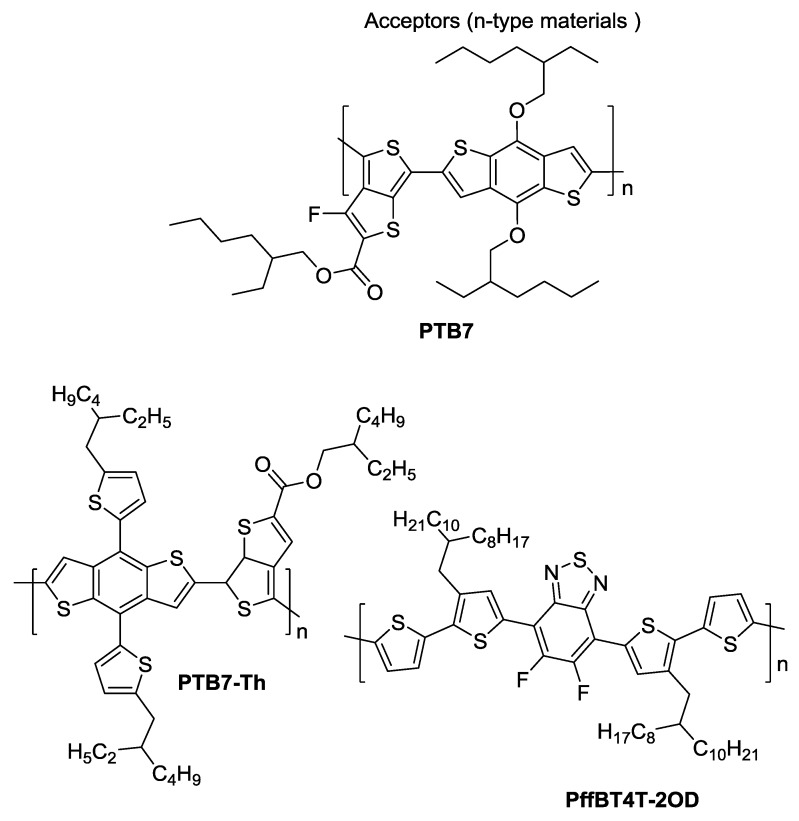
Chemical structures of the polymer donors used in OPVs in this review.

Figure 4 depicts the chemical structure of these three very popular “post-P3HT” conjugated polymers used in OPVs. In this review only devices based on these three polymers (PTB7 [72], PTB7-Th and PffBT4T-2OD) will be discussed.

Buckminsterfullerene C_60_ was the first fullerene used in an OPV device, in the seminal work by Sariciftci et al. [73], where these authors report the photo-induced electron-transfer from a conducting polymer to C_60_. However, the very low solubility of C_60_ in common organic solvents makes it very difficult to process and therefore, soon after its introduction in the OPV field, the strategy of functionalizing C_60_ with solubilizing moieties was adopted. For this reason the fullerenes PC_61_BM [74,75] and its analogue PC_71_BM [76] soon emerged as the two most widely used electron accepting materials in organic photovoltaics (OPVs). These two fullerenes, PC_61_BM and PC_71_BM, are now utilized as reference acceptors for all kinds of other fullerene acceptors, because of their good solubility, high electron mobility and high chemical stability. A key difference between PC_61_BM and PC_71_BM is the ellipsoidal shape of the latter, as compared to the more spherical C_60_ molecule [77]. The lower symmetry and more extended conjugation of C_70_ enables energetic transitions that are forbidden in C_60_, leading to a broader photo-absorption profile in the visible region of the solar spectrum [78]. This allows increased photon harvesting and a potentially higher photocurrent for devices using PC_71_BM rather than PC_61_BM, an important attribute that has brought the C_70_ analogue to the forefront of OPV research (despite its higher cost).

**Figure 5:**
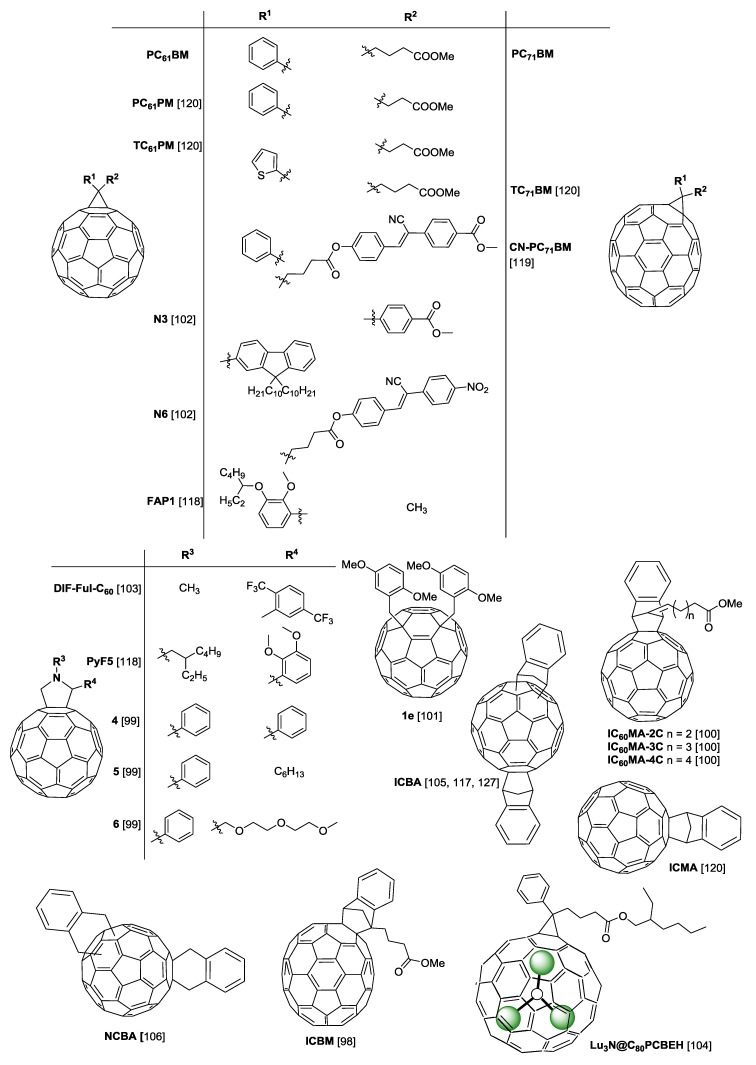
Chemical structures of the fullerene acceptors tested in OPVs in this review.

Although PC_61_BM and PC_71_BM are the most commonly used fullerenes in organic solar cells, several other modified fullerenes such as those depicted in Figure 5 are found in literature with specific characteristics and have been assessed as acceptors in BHJs. This will be discussed below in combination with the corresponding polymers depicted in Figure 4.

As mentioned above, the *V_oc_* of a polymer:fullerene solar cell increases when the energy difference between the LUMO level of the fullerene and the HOMO level of the polymer increases (Figure 3a). Therefore, using fullerenes with a higher LUMO level results in devices with higher *V_oc_* values. A common synthetic approach used to increase the LUMO level of fullerenes consists in adding more addends to the fullerene cage in order to obtain fullerene multiadducts. Such strategy reduces the number of double bonds and the level of conjugation in the fullerene molecule and therefore increases the corresponding LUMO level. The fullerene bisadduct 1′,1″,4′,4″-tetrahydro-di[1,4]methanonaphthaleno[5,6]fullerene-C_60_, commonly known as ICBA (Figure 5), has been the most popular bisadduct used in solar cells. However, as will be seen throughout this review, most often, although fullerene bisadducts such as ICBA produce devices with higher *V_oc_* values, the corresponding PCE values are lower than those of reference devices with PC_71_BM and PC_61_BM. The reasons for this are that fullerene multiadducts usually have much lower electron mobilities than monoadducts and this impacts the device’s *J_sc_* and *FF* adversely. Furthermore, these multiadducts are typically mixtures of several isomers and the small differences in the LUMO levels of these isomers contribute to increase the probability of exciton recombination and thus further decrease *J_sc_* and *FF*.

## 3. Additives

The processing conditions used in the preparation of a BHJ play a crucial role in the corresponding morphology and this also plays a critical role on the corresponding device energy efficiency. The use of additives [79,80] is by far the most popular processing methodology used to optimize the morphology and increase device performance in OPV devices using the small band gap copolymer systems PTB7, PTB7-Th and PffBT4T-2OD. This methodology consists in adding, before BHJ thin film deposition, a small amount of a high boiling point additive to the photovoltaic ink solution, typically 3 vol %. The most popular additives include 1,8-diiodooctane (DIO), 1-chloronaphthalene (CLN) and 1,8-octanedithiol (ODT), among others. In many instances, additives can more than duplicate the PCE of the corresponding devices.

Despite the several studies performed using additives to improve the morphology of OPVs, these have been largely driven by empiricism as there is still a lack of fundamental scientific understanding of the relationship between the processing additive used, the resultant BHJ morphology and the corresponding device efficiency.

The primary role of additives such as DIO was, until recently, the object of some debate in the field. In a 2011 highly cited work, Lou et al. [81] used small-angle X-ray scattering (SAXS) to study the structure of PC_71_BM in chlorobenzene (CB), in a concentration of 15 mg·mL^−1^ and found that the addition of a small amount of DIO (3 vol %) to CB change the scattering signal of the fullerene solution. Their model-based analysis of the scattering data led them to interpret these results as evidence that the initial CB solution, without DIO, contained PC_71_BM aggregates and that after the addition of DIO these were completely eliminated from solution due to an increased solubility of PC_71_BM in the CB:DIO solution. This elimination of PC_71_BM agglomerates was hypothesized by Lou et al. to be the reason for the efficiency improvement in the OPV devices, when spin-coated with the polymer PTB7, as a result of the improved intercalation of the fullerene.

**Figure 6:**
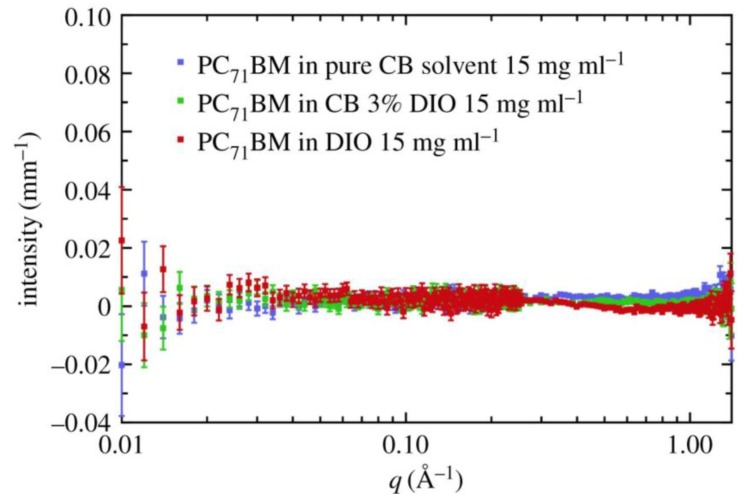
SANS solution data for PC_71_BM at a concentration of 15 mg mL^−1^ in pure CB, CB with 3 vol % DIO and also in pure DIO. Reprinted with permission from Ref. [83].

However, some more recent studies have cast considerable doubts upon this work by Lou et al. In 2014, Burgués-Ceballos et al. [82] reported that the solubility of PC_71_BM in CB was 207 mg·mL^−1^, that is, much higher than the concentration of the solution (15 mg·mL^−1^) in which Lou et al. [81] reported the presence of PC_71_BM agglomerates. Very recently Bernardo et al. [83,84] carried out a full study aiming to understand the solution state of PC_71_BM in CB and CB with 3% DIO. Using both SAXS and SANS, these authors have shown that there is no change in the dissolution state of PC_71_BM upon the addition of DIO and in fact as shown in Figure 6, there is no aggregation of PC_71_BM in solution with or without the additive, contrary to the analysis previously reported by Lou et al.

Furthermore, by studying the drying process of the spin-coated films using optical interferometry, these authors concluded that the high boiling point additive DIO plays its role by staying in the film after the spin coating process has finished. This allows increasing the molecular mobility of the DIO solvated fullerene on a much longer timescale than that of the film without DIO.

**Figure 7:**
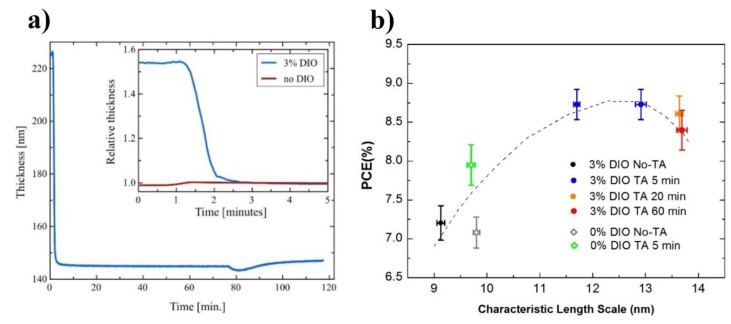
(**a**) Dynamic spectroscopic ellipsometry data for the isothermal annealing of a PffBT4T-2OD:PC_71_BM blend film annealed at 100 °C. The heating stage was at 100 °C at time *t* = 1 min. The inset shows a zoomed in region at the beginning showing the dramatic drop in thickness within the first minute of being at temperature; (**b**) Correlation between PCE and characteristic length scale for samples processed in different ways, that is, with/without DIO and with/without annealing. Reprinted with permission from ref. [39].

A similar conclusion was reached by Zhang et al. [39] studying PffBT4T-2OD:PC_71_BM based devices. Using spectroscopic ellipsometry, these authors showed that after spin-coating, a BHJ film processed using DIO is much thicker than a BHJ film processed without DIO, which clearly indicates that DIO largely remains in the film. Furthermore, upon annealing, the thickness of the film processed with DIO decreases continuously due to DIO evaporation until reaching a thickness similar to the film processed without DIO (Figure 7a). Additionally, using SANS, these authors demonstrated that in these PffBT4T-2OD:PC_71_BM devices, DIO improves the efficiency as a result of the coarsening of the fullerene phase domains in the BHJ film induced by the transient plasticisation of the film during drying (Figure 7b).

## 4. Devices Based on PTB7

PTB7 (Figure 4) is a copolymer containing an alternating electron rich benzodithiophene (BDT) unit and an electron deficient thienothiophene (TT) unit [72]. In this chapter Section 4.1 presents seminal research works on the improvement of the BHJ morphology and efficiency of PTB7:PC_71_CM (or PC_61_BM) solar cells, discussing afterwards some of the most recent developments in this topic. Section 4.2 addresses the most recent and relevant studies involving BHJs of PTB7 with other fullerenes. In the end of this chapter, Table 1 summarizes the most relevant and most recent device efficiencies obtained with PTB7-based solar cells.

### 4.1. PTB7 Devices with PC_61_BM and PC_71_BM

The use of the polymer PTB7 in BHJ solar cells was first reported in 2010 in a seminal work by Liang et al. [85]. The best devices reported in this work were prepared with a D:A mass ratio of 1:1.5, using CB as solvent with 3 vol % of DIO as additive and exhibited a PCE of 7.40% being, at the time, the first polymer solar cell showing a PCE over 7%. Zhou et al. [86] reported short after a PCE to 7.94% after treating similar devices with methanol. Liu et al. [71] showed that it was possible to push the PCE values to 8.5% using a PTB7 with high molecular weight (*M_n_*) of 128 kg/mol and a low polydispersity (PDI) of 1.12. He et al. [87] fabricated devices, in the normal and inverted geometries, using the polymer PFN (poly[(9,9-bis(3′-(N,N-dimethylamino)propyl)2,7-fluorene)-alt-2,7-(9,9-dioctylfluorene)]) as a surface modifier to optimize the electrode work-function and increase the photocurrent. The reported PCE for the conventional and inverted geometries were respectively 8.24% and 9.15%.

In the last three years, the optimization of the efficiency of PTB7:PC_71_BM (or PC_61_BM) devices progressed actively by improving the morphology of the BHJ.

Jhuo et al. studied the effect of new additive 1-naphthalenethiol (SH-na) on the morphology of PTB7:PC_71_BM BHJs. This additive was tested for bulk and surface morphology control, via a conventional spin-casting process and a novel SH-na solution dipping treatment [61]. Five different sets of devices were tested, namely: (i) reference device without additive; (ii) reference device with the conventional spin-cast DIO additive; (iii) device with spin-cast SH-na additive; (iv) device without spin-cast additive but with solution dipped SH-na additive and (v) device with both spin-cast SH-na additive and solution dipped SH-na additive. The top performing devices were those prepared by spin-casting SH-na additive and solution dipped SH-na additive and these exhibited a PCE of 8.42%, a Voc of 0.79 V, a *J_sc_* of 15.7 mA·cm^−2^ and a *FF* of 0.70, which contrasted deeply with the corresponding values for a standard device without additive that were, respectively, 3.68%, 0.70 V, 11.4 mA·cm^−2^ and 0.46.

The BHJ morphology and photovoltaic performance of PTB7:PC_71_BM devices was studied upon adding a series of bromine-terminated additives with different chain lengths in the range from 1,2-dibromoethane to 1,8-dibromooctane [88]. Despite the chemical similarity between the different additives, they were found to produce very different morphologies. Compared to devices without additive (PCE of 3.57%), devices processed with the additive 1,4-dibromobutane originated the highest performances, although these were modest (PCE of 5.53%). Interestingly, among all the additives tested 1,4-dibromobutane exhibited the closest solubility parameter to that of the classical DIO additive.

Others [89] tested the effect of additives and additive mixtures (including DIO, CLN and ODT) in the performance of PTB7:PC_61_BM devices prepared from solutions using chloroform as host solvent. The best devices reported had a PCE of 6.41% and were obtained using a mixture of DIO and CLN as additive.

The use of a new solvent additive—diphenyl ether (DPE)—that facilitates the fabrication of thick (180 nm) PTB7:PC_71_BM BHJ layers and that, compared with additive-free devices, triplicates the PCE from 1.75% to 6.19% due to an increase in the crystallization of PTB7 that facilitates charge transport over longer distances, was reported by Zheng et al. [90]. Very recently, the same team reported [91] a novel binary solvent additive of DPE:DIO that, when added in the proportion of 2 vol % DPE + 3 vol % DIO to a CB ink solution, boosts the efficiency of inverted PTB7:PC_71_BM devices to over 9.5%, being this among the best results ever reported in the literature for PTB7-based devices. According to the authors, this improvement was due to the synergistic effect of both additives, with DPE promoting the crystallization of PTB7 and DIO favouring the phase separation between PTB7 and PC_71_BM. This work highlighted the great potential of binary solvent additives for improving the performance of OPVs.

The effect of diiodoalkane additives with variable alkyl chain length, from 1,4-diiodobutane to 1,10-diiodododecane, on the performance of inverted PTB7:PC_71_BM devices was studied by Zhao et al. [92]. Compared with standard devices prepared from CB ink solutions without additive (PCE of 2.3%), all the diiodoalkane additives tested improved considerably the efficiency. However, the best performing devices with a PCE of 8.84% were still obtained using the classical DIO (3 vol %), which shows that DIO has the optimal alkyl chain length to maximize the performance of these devices. Impedance spectroscopy results indicate that DIO maximizes the charge transfer from the BHJ to the electrode by minimizing the recombination rate at the donor/acceptor interface and increasing the charge carrier lifetime.

Formic acid (FA) was presented by Li et al. [93] as a novel additive capable of improving the efficiency of PTB7:PC_71_BM solar cells when used in combination with DIO in ink solutions using CB as host solvent. The device prepared using a solution prepared with 6 vol % FA and 3 vol % of DIO showed the best PCE of 9.04% compared with a PCE of 7.25% for the control device with 3 vol % DIO and no FA. This increase in PCE observed upon the addition of FA was due to a dramatic increment of *J_sc_* from 14.57 mA·cm^−2^ in the control device, to 24.11 mA·cm^−2^ in the devices with FA.

Chen et al. [94] introduced o-chlorobenzaldehyde (CBA) as a new solvent additive. PTB7:PC_71_BM devices were tested with different amounts of CBA and different BHJ thicknesses (100, 200 and 300 nm) and simultaneously standard devices without additive and devices with the standard 3 vol % DIO were also produced to be used as reference devices. The best devices exhibited a PCE of 3.87% without any additive and a PCE of 7.53% with DIO. However, the most efficient devices were those processed with CBA (5 vol %) and with a BHJ thickness of 100 nm, which achieved an impressive PCE of 9.11%. Charge carrier transport properties, determined with space-charge-limited current (SCLC) measurements, showed that the best devices processed with CBA had more ideal hole mobility (μ_h_) and electron mobility (μ_e_) than the other best devices: without any additive, μ_h_ = 7.3 × 10^−5^ cm^2^V^−1^s^−1^, μ_e_ = 5.9 × 10^−5^ cm^2^V^−1^s^−1^ and μ_h_/μ_e_ =1.24; with DIO, μ_h_ = 8.4 × 10^−5^ cm^2^V^−1^s^−1^, μ_e_ = 1.6 × 10^−4^ cm^2^V^−1^s^−1^ and μ_h_/μ_e_ =0.53; with CBA, μ_h_ = 1.3 × 10^−4^ cm^2^V^−1^s^−1^, μ_e_ = 4.0 × 10^−4^ cm^2^V^−1^s^−1^ and μ_h_/μ_e_ = 0.33. Transient photovoltage (TPV) measurements revealed that devices processed with CBA displayed longer charge carrier lifetimes and shorter charge extraction times than devices processed with DIO or without additives. These are consistent with the higher *FF* and *J_sc_* observed in the devices with CBA additive. Besides producing devices with higher efficiencies, this work has also shown that CBA, when compared to DIO, has the additional advantage of possessing a much lower boiling point (b.p.= 212 °C vs. b.p.= 350 °C for DIO) but still sufficiently higher than CB (b.p. = 132 °C). Thus, contrary to DIO, which requires high-vacuum post-treatments in order to be completely removed from the films, CBA evaporates during thin film processing, being therefore compatible with roll-to-roll processing.

Another topic related to the BHJ, which has attracted some attention in recent years, is the morphological and lifetime stability of the PTB7-based solar cells.

The effect of two different host solvents (CB and ortho-xylene) containing 3 vol % DIO additive, on the morphology, efficiency and stability of inverted PTB7:PC_61_BM devices was studied by Ciammaruchi et al. [95]. Devices processed with both solvents exhibited similar PCEs (7.54% with CB and 7.05% with ortho-xylene). Lifetime stability studies suggests that a complete removal of solvent traces as well as a proper UV filtering can largely improve the stability of cells processed with ortho-xylene and therefore eliminate the halogenated solvents from the process.

Dkhil et al. [96] reported that, depending on the fullerene size, there is a large difference in the morphological stability of PTB7:fullerene blends as a function of temperature. After 1 h of annealing at 140 °C, the PTB7:PC_61_BM films showed the formation of fullerene crystals that increase in size and number after further annealing for 1 day. In contrast, PTB7:PC_71_BM blends did not show any fullerene crystals visible by optical microscopy after one day thermal annealing at 140 °C. This clearly shows that there is a strong improvement in thermal stability of PTB7 polymer blends using the larger fullerene derivative PC_71_BM. Furthermore, PTB7 devices with PC_71_BM exhibited a remarkably high temperature stability during permanent annealing at 140 °C for several days, which can be directly related to the intrinsically stability of the polymer blend.

The UV-induced degradation of PTB7:PC_71_BM solar cells in an inert atmosphere was studied by Bartesaghi et al. [97] who concluded that this degradation is due to a photochemical reaction that requires the presence of both PTB7 and PC_71_BM. However, the exact mechanism of the photochemical degradation could not be determined.

### 4.2. PTB7 Devices with Other Fullerenes

A new fullerene acceptor (ICBM in Figure 5) was synthesized by He et al. using the cyclic addition reaction between an indene derivative (methyl 4-(1H-inden-3-yl) butanoate) and C_70_ [98]. Cyclic voltammetry results showed that the LUMO level of ICBM is 0.07 eV higher than that of PC_71_BM. PTB7:ICBM devices, as well as PTB7:PC_71_BM reference devices, were prepared using polymer:fullerene mass ratio of 1:1.5 and 3 vol % DIO as solvent additive to optimize the device morphologies. Compared to reference devices, ICBM-based devices showed higher *V_oc_* (0.79 vs. 0.74 V), as expected due to the higher LUMO level of ICBM, as well as higher *J_sc_* (15.4 vs. 14.2 mA·cm^−2^). However, the FF of PTB7/ICBM devices was slightly lower than that of PTB7/PC_71_BM. Nevertheless, the final PCE of PTB7/ICBM devices (6.67%) was higher than that of PTB7/PC_71_BM (6.30%) due to the improvement of both *J_sc_* and *V_oc_*.

Novel [60] fulleropyrrolidine derivatives were synthetized by Karakawa et al., where the replacement of the methyl butyrate group at C-2 on the pyrrolidine ring with phenyl, hexyl and 2,5,8-trioxanonyl substituents lead to a series of *N*-phenyl derivatives 4, 5 and 6, respectively (Figure 5) [99]. The devices were made with inverted structure: ITO/PFN/BHJ/MoOx/Al, in which PFN is used as a cathode interlayer [87]. The 4-based device exhibited a higher *J_sc_* compared with other devices, whereas the devices using C-2 alkylated 5 and 6 showed higher *V_oc_* values than the 4-based and PC_61_BM-based devices, probably due to their slightly higher LUMO energy levels. Overall, the device using the PTB7/4 blend film exhibited the highest PCE of 7.34% among these devices. It is worth noting that the PCE values of the PTB7/4 and PTB7/5 devices are both higher than that of the reference PTB7/PC_61_BM device (7.03%).

Tseng et al. synthesized three IC_60_MA derivatives namely: IC_60_MA-2C, IC_60_MA-3C and IC_60_MA-4C (Figure 5) with higher LUMO level than PC_61_BM and tested them in PTB7-based devices. As expected, the devices of PTB7 with ICMA derivatives all exhibited higher *V_oc_* than the reference device of PTB7:PC_61_BM [100]. However, the best PCE was still obtained with the reference PTB7:PC_61_BM devices (6.8%), while the devices with IC_60_MA-2C, IC_60_MA-3C and IC_60_MA-4C displayed PCEs of 6.0%, 5.1% and 6.5%, respectively.

Huang et al. studied 10 newly synthesized 1,4-fullerene bisadducts (methoxylated 1,4-bisbenzyl fullerene adducts) [101], in which the two substituents are located at the “para” positions of a six-membered ring on the fullerene cage. These new fullerenes have a smaller π-conjugated system with reduced symmetry and, therefore, their LUMO level is slightly higher compared with 1,2-fullerene bisadducts. These fullerenes were all initially tested with the standard P3HT polymer. Later, only the most successful (1e—Figure 5) was tested with PTB7 using a polymer:fullerene mass ratio of 1:1.5 and DIO as additive. For comparison, reference devices with PTB7:PC_61_BM were also prepared. Although the PTB7:1e device showed a much higher *V_oc_* (0.83 vs. 0.76 V) and a slightly higher *J_sc_* (12.3 vs. 12.1 mA·cm^−2^) than the reference device, its overall PCE was slightly lower than in the reference device (5.4 vs. 5.9%) due to a considerably lower *FF* (53.3 vs. 64.4%), which may be due to a less efficient crystallization of derivative 1e compared to PCBM, changing the resultant BHJ morphology.

Two new fullerene acceptors, namely N3 and N6 in Figure 5, were synthetized by Nagarjuna et al. and tested in solar cells based on PTB7. The cyclopropane ring in N3 is attached to two aryl rings: one aryl ring consists in fluorene with two long decyl chains, which increase the solubility of the molecule in organic solvents and the other aryl ring is a methyl benzoate substituent, that has an electron withdrawing CO_2_CH_3_ group (EWG), which increases the electron accepting nature of the fullerene [102]. The fullerene N6 differs from N3 in that the methyl benzoate substituent is replaced by a long substituent containing NO_2_ and CN groups, which are EWG. The highest PCE (4.1%) of devices with an inverted architecture was achieved with the system PTB7:N3 due to a slightly higher *J_sc_* and *V_oc_*. The poor shunt resistance in PTB7:N6 solar cells indicates more recombination of charge carriers in these devices. Lower solubility of N6 in 1,2-dichlorobenzene (o-DCB) may cause reduced donor-acceptor interface for excitons to be broken up into charges and insufficient percolation paths for charges to get collected at the electrode in PTB7:N6 films, resulting in higher recombination than in PTB7:N3 devices. On the other hand, series resistances were found to be 16.9 and 13.7 Ω·cm^2^ for PTB7:N3 and PTB7:N6, respectively. A higher value of series resistance in the PTB7:N3 device is possibly due to a higher thickness of the film (85 nm) compared to that of PTB7:N6 blend films (70 nm) [102]. In a later work, the same authors synthetized trifluoromethyl derived fulleropyrrolidine (DIF-ful-C_60_ in Figure 5) [103] fullerenes in which the attached −F and −CF_3_ groups enhance the electron accepting character of the resultant fullerene due to the high electron deficient property of the ligands. The inverted devices with PTB7:DIF-ful-C_60_ displayed a slightly higher efficiency than the control device of PTB7:PC_61_BM (6.8 vs. 6.2%) due to a higher *V_oc_* (0.82 vs. 0.70 V) and a higher *J_sc_* (−15.97 vs. −15. 40 mA·cm^−2^).

In a highly original work, the impact of the endohedral fullerene Lu_3_N@C_80_PCBEH (Figure 5) on the morphology and efficiency of PTB7-based devices was tested by Roehling et al. [104]. This endohedral fullerene has a lower electron affinity than standard fullerenes, which can raise the *V_oc_* of photovoltaic devices. However, the PCE values obtained with standard devices processed with a o-DCB solution without additives were very poor (PCE of 0.4%) and, surprisingly, deteriorate further with the addition of additives. A morphological study showed that the poor performance results from a poor miscibility between PTB7 and Lu_3_N@C_80_PCBEH and in films with DIO added, the fullerene was observed to strongly aggregate into micrometre sized crystals.

Ternary polymer solar cells involving BHJs of PTB7 with PC_71_BM and other fullerene have also been investigated.

ICBA (Figure 5) was used by Cheng et al. [105] as an electron-cascade acceptor material in ternary blend devices of PTB7:PC_71_BM:ICBA. Due to the higher LUMO level of ICBA relative to PC_71_BM, the addition of ICBA leads to an increase in the *V_oc_*. The best performing devices were those with a PC_71_BM:ICBA ratio of 85:15 and exhibited a PCE of 8.24% compared to 7.35% for standard binary blend devices without ICBA.

Very recently Ma et al. [106] added a dihydronaphthyl-based C_60_ bisadduct (NCBA in Figure 5) to a normal PTB7:PC_71_BM BHJ. NCBA has a LUMO between the LUMOs of PTB7 and PC_71_BM and, therefore, plays a bridging role allowing the acceptor energy level to be tuned by changing the ratio of NCBA:PC_71_BM in the blend acceptor material. The best devices were obtained with a 15% mass ratio of NCBA and exhibited a PCE of 9.85%, compared with a PCE of 8.57% for the reference device without NCBA.

## 5. Devices Based on PTB7-Th

PTB7-Th (Figure 4) possesses a conjugated backbone structure identical to PTB7, only differing on the nature of the organic ligand connected to the benzodithiophene (BDT) unit: the ether group in PTB7 is replaced by a thiophene group in PTB7-Th. Due to this chemical modification, PTB7-Th can sustain higher temperatures than PTB7 without suffering decomposition [107]. In this chapter, Section 5.1 discusses the most relevant studies performed in the optimization of the BHJ morphology and efficiency of PTB7-Th:PC_71_BM (or PC_61_BM) devices. Later, Section 5.2 addresses devices incorporating BHJs of PTB7-Th with other fullerenes. A compilation table, Table 2, is presented at the end of this chapter summarizing the most relevant and recent device efficiency results obtained with OPVs based on PTB7-Th.

### 5.1. PTB7-Th Devices with PC_61_BM and PC_71_BM

PTB7-Th synthesis and testing in polymer solar cells was first reported by Zhang et al. [108] in 2014. A PCE of 9.0% was achieved in standard devices containing PTB7-Th:PC_71_BM in the ratio 1:1.5 and spin-coated from o-DCB solution with 3 vol % DIO. Devices processed with 1 vol % DIO and 5 vol % DIO were also tested but produced worse PCE results.

Huang et al. studied the effect of the solvent additive DIO on the BHJ morphology and efficiency of PTB7-Th:PC_71_BM devices with inverted architecture, using PEIE (Polyethylenimine ethoxylated) as interfacial layer on top of ITO [109]. The device prepared from a pure CB solution, that is, without additive, shows an efficiency of 6.4% with a *J_sc_* of 16.2 mA·cm^–2^, a *V_oc_* of 0.80 V and *FF* of 49%. Incorporating 3 vol % of DIO, the efficiency improves to 9.5%, corresponding to *J_sc_*, *V_oc_* and *FF* values of 18.1 mA·cm^–2^, 0.79 V and 66%, respectively. AFM and TEM studies confirm the effectiveness of DIO in improving the dispersion of PC_71_BM: the large aggregates ≈100 nm in diameter observed in films prepared without DIO are suppressed in films prepared with DIO [109].

A binary solvent additive made of DIO:N-methyl-pyrrolidine (NMP) (1.5%:1.5%) was employed by Wan et al. [110] to improve the PCE of PTB7-Th:PC_71_BM devices from 8.2% (without additive) and 9.5% (with 3 vol % DIO) to 10.8%. Resonant Soft x-Ray Scattering analysis showed that, although all the BHJs exhibit similar domain sizes, the BHJ processed with DIO:NMP showed higher phase purity than the BHJs with single additives or without additives, which facilitates the charge transport and reduces charge recombination leading to the higher PCE observed.

Fan et al. [111] introduced a solid (melting point, m.p. = 125 °C) fluorescent inhibitor (1-bromo-4-nitrobenzene) into the PTB7:PC_71_BM BHJs to improve device efficiency. All the devices were spin-coated from a CB solution with 3 vol % DIO and the best devices were obtained with 15 wt % of 1-bromo-4-nitrobenzene and exhibited a PCE of 8.95% compared to 7.58% for a reference device without 1-bromo-4-nitrobenzene. The increase in PCE was attributed to an improvement in charge transport and dissociation.

The effect of different polymer:fullerene blend mass ratios (1:0.5; 1:1; 1:1.5; 1:2 and 1:3) on the photovoltaic performance of PTB7-Th:PC_71_BM devices, with standard architecture, was investigated by Komilian et al. [62]. No additives were used and the surface of the BHJ was washed with ethanol before top electrode deposition. The best devices with a PCE of 9.38% were obtained with a D:A mass ratio of 1:2.

Xiao et al. [7] reported highly efficient ternary cells based on PTB7-Th:CO_i_8DFIC (Acceptor A1):PC_71_BM (Acceptor A2), where COi8DFIC is a non-fullerene small molecule acceptor. The LUMO for PTB7-Th (−3.12 eV), PC_71_BM (−3.67 eV) and CO_i_8DFIC (−3.88 eV) show a stepwise alignment and therefore PC_71_BM facilitates electron transfer from PTB7-Th to CO_i_8DFIC. The mass ratio between PTB7-Th and A1+A2 was fixed to 1:1.5, while the content of A2 in acceptors was varied from 0 to 100%. The best devices were obtained with (D:A1:A2) mass ratios of (1:1.05:0.45) and produced a remarkable PCE of 14.08%. These ternary cells combine the advantage of fullerene acceptors (high μ_e_) and non-fullerene acceptors (strong visible or NIR absorption). Charge carrier mobilities were measured using the SCLC method. Compared with the binary blend film PTB7-Th:CO_i_8DFIC (μ_h_ = 6.98 × 10^−4^, μ_e_ = 3.89 × 10^−5^ and μ_h_/μ_e_ = 18), the ternary blend film PTB7-Th: CO*_i_*8DFIC:PC_71_BM (1:1.05:0.45) showed a similar μ_h_ and a much higher μ_e_, which resulted in a decrease in the ratio μ_h_/μ_e_ from 18 to 1.3 (μ_h_ = 6.35 × 10^−4^, μ_e_ = 4.80 × 10^−4^ and μ_h_/μ_e_ = 1.3). As a result of the improved electron transport and more balanced charge transport in the active layer containing PC_71_BM, *J_sc_* and *FF* increased.

Jagadamma et al. [112] investigated the effect of ex-situ thermal annealing on the BHJ morphology of PTB7-Th:PC_71_BM devices and on the corresponding efficiency. Devices with an inverted architecture were used and the thermal annealing was performed over a range of temperatures ranging from room temperature to 150 °C. To exclude any negative influence from the possible degradation of charge selective layers and metal contacts, the ex-situ thermal annealing was applied only to the BHJ, that is, before metal anode deposition and not to the complete device. The unannealed devices displayed the highest PCE (9.1%) and this decreased gradually with the increase in thermal annealing temperature until it reached its lowest value (6.9%) for devices that had been annealed at 150 °C. A morphological analysis using AFM showed that the BHJ morphology coarsens with increasing temperature and large scale phase separation is observed in BHJ annealed at 150 °C which partially supresses exciton dissociation and increases recombination losses with the consequent drops observed in *J_sc_*, *FF* and in the PCE.

The effect of thickness variation of the BHJ (from 62 nm to 307 nm) on the photovoltaic performance of PTB7-Th:PC_71_BM devices, with both standard and inverted architectures, was studied by Kobori et al. [113]. In both device architectures, it was observed that *FF* decreased continuously with increasing the BHJ thickness. By contrast, the *J_sc_* increases with the BHJ thickness but not monotonically. Furthermore, the *J_sc_* of inverted devices were systematically higher than those of normal devices with identical thickness. In terms of overall PCE, for lower BHJ thickness (<100 nm) inverted devices perform considerably better than normal devices. However, for higher thicknesses the difference between the PCE of standard and inverted devices tends to fade away. The optimized devices were obtained in standard geometry with a BHJ thickness of 116 nm and a corresponding PCE of 9.25% and in inverted geometry with a BHJ thickness of 76 nm and a corresponding PCE of 10.4%. These results were explained by the difference of the simulated optical intensity distribution in the devices.

In a similar work, Zang et al. [59] also studied the effect of BHJ thickness (70, 90, 120, 180 and 270 nm) and polymer:fullerene (D:A) mass ratio (1:1.5 and 1:3) on the performance of PTB7-Th:PC_71_BM devices with an inverted architecture. For devices with a D:A mass ratio of 1:1.5, a BHJ thickness of 90 nm produced the highest efficiency with a PCE of 9.68%, a *V_oc_* of 0.80 V, a *J_sc_* of 16.55 mA·cm^−2^ and a *FF* of 71%. Although the devices with a thicker BHJ (270 nm) displayed a considerably higher *J_sc_* (19.70 mA·cm^−2^), in agreement with the previous work by Kobori et al. [113], the corresponding PCE was lower (7.79%) due to a drastic drop in *FF* (50%) which offset the improved light absorption resultant from using thicker films. This decrease in *FF* was attributed to the less efficient charge carrier transport and dissociation in thicker films, due to the relatively low electron mobility in the BHJ. To improve the μe of the BHJ, the authors also tested devices in which they changed the D:A mass ratio to 1:3. In this case they observed that for thick devices with a BHJ thickness of 270 nm, the PCE was higher (8.15%) than the PCE of the corresponding devices with a D:A mass ratio of 1:1.5 (7.79%).

Other researchers studied the effect of different processing treatments on bulk and interfacial disorder in inverted PTB7-Th:PC_71_BM devices [114]. Devices fabricated with and without DIO showed PCEs of 8.3% and 3.8%, respectively.

The lifetime stability and degradation of the PTB7-Th based solar cells has also been a topic of interest in recent years.

Pearson et al. [115] measured the stability of reference PTB7-Th:PC_71_BM devices, with both standard and inverted architectures, over 70 h in an atmospheric chamber under continuous dry nitrogen flow, with typical oxygen and moisture levels of <5 ppm and <30 ppm respectively. Although devices with normal architecture exhibited a larger initial efficiency (PCE of 7.3% on average, vs. PCE of 6% for inverted cells), a PCE drop of more than 60% was observed in both device structures after only 24 h of light soaking under nitrogen. These results strongly suggest that BHJ degradation is driven by light-mediated processes rather than high levels of oxygen and moisture exposure. The large PCE reduction observed in both device configurations was mostly due to a large overall reduction in *J_sc_*. Two different strategies were proposed for improving the stability of both standard and inverted devices. For standard devices, DIO was replaced by o-DCB as a co-solvent. Although the PCE of these new devices was slightly lower (6.5%), the device stability was greatly improved, which highlighted the detrimental stability effects associated with the use of DIO and previously identified by others in different BHJ systems. For inverted devices, placing a UV filter in front of the devices was also found to reduce considerably the extent of PCE degradation.

**Figure 8:**
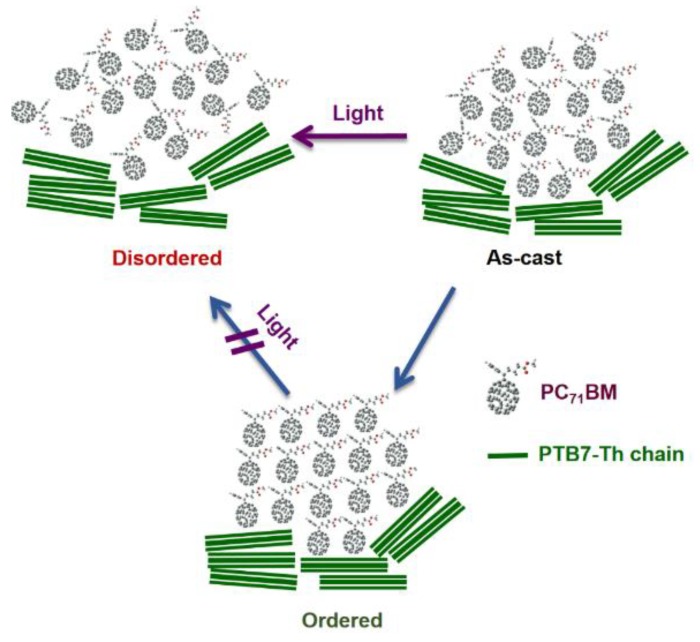
Schematic of two possible paths for the nanomorphology evolution in the PC_71_BM acceptor phase: in the top one light induces disorder and in the bottom one light induced disorder is prevented in a highly crystalline configuration of the PC_71_BM molecules. Reprinted with permission from ref. [116].

Liu et al. [116] demonstrated that in encapsulated devices, completely isolated from moisture and oxygen, the absorption of high energy UV photons by the PCBM molecules leads to a degradation of exciton diffusion and charge mobility in the fullerene phase. The μ_e_ measured by the SCLC method decreased by four orders of magnitude, from 2.43 × 10^−3^ to 2.16 × 10^−7^ cm^2^V^−1^s^−1^ and the μ_h_ decreased by less than one order of magnitude, after 45 h of AM 1.5G non-UV-filtered 1-sun illumination. Transient Absorption (TA) measurements combined with UV wavelength cut-off degradation experiments suggest that the burn-in may be triggered by a spin flip at the donor/acceptor interface, leading to the formation of PC_71_BM triplet anions and the accumulation of electrostatic potential energy. The release of this excess electrostatic potential energy promotes a disordering of the weakly bonded nanomorphological order, mostly in the PC_71_BM domains near the donor/acceptor interface. To circumvent this problem, the authors proposed a strategy that consisted in replacing CB by DCB as master solvent, removing the DIO from the processing solution, replacing ZnO buffer layer (an oxidant source) by PFN and applying a thermal annealing treatment, prior to light soaking, to increase the crystallinity of the PC_71_BM domains in the BHJ (Figure 8). Using this strategy, these authors were able to produce organic cells that were simultaneously highly efficient and very stable.

### 5.2. PTB7-Th Devices with Other Fullerenes

Huang et al. [117] studied the BHJ morphology, photophysics and performance of inverted devices based on PTB7-Th blended with the indene-C_60_ bisadduct ICBA (Figure 5) and correlated it with similar devices using the two classical fullerenes PC_61_BM and PC_71_BM. Although devices based on ICBA achieved, as expected, a higher *V_oc_* (1.0 V) than devices based on PC_61_BM and PC_71_BM (0.81 V and 0.80 V respectively) consistent with the higher LUMO of ICBA, the corresponding efficiency was lower (average PCE of 7.1% for devices with ICBA compared with 7.6% and 9.4% for devices based on PC_61_BM and PC_71_BM, respectively). Comparing the *J_sc_* values, PTB7-Th devices based on PC_71_BM have a much higher *J_sc_* (17.7 mA·cm^−2^) than similar devices based on PC_61_BM (14.6 mA·cm^−2^) and ICBA (13.3 mA·cm^−2^). This difference is largely explained by the fact that, in the visible region of the spectrum, PC_71_BM has a much higher absorption coefficient than both PC_61_BM and ICBA. ICBA BHJs are also characterized by lower polymer crystallinity, smaller domain sizes and better-mixed phases, which promotes a faster geminate recombination as revealed by transient absorption (TA) measurements. Overall this results in a poorer device performance of the ICBA-based devices.

The carrier transport loss mechanism in PTB7-Th BHJs based on the amorphous fullerene acceptor PyF5, as well as on the semi-crystalline fullerene acceptors FAP1 and PC_61_BM (see chemical structures of PyF5 and FAP1 in Figure 5) were studied by Zhang et al. [118]. The PCE of optimized PyF5 and FAP1 based devices (6.5% and 6.1% respectively) is slightly lower than that of optimized PC_61_BM based devices (7.3%), owing to the slightly lower fill factor and *J_sc_*. Interestingly, optimized devices based on the fullerenes PyF5 and FAP1 require a higher fullerene mass loading (D:A = 1:2) than the optimized device based on PC_61_BM (D:A = 1:1.5). The charge carrier transport properties of pristine fullerenes and PTB7-Th:fullerene blends were determined with SCLC measurements. Although the three pristine fullerenes have very similar μ_e_ values (PyF5 = 4.0 × 10^−3^ cm^2^V^−1^s^−1^; FAP1 = 4.4 × 10^−3^ cm^2^V^−1^s^−1^; PC_61_BM = 4.5 × 10^−3^ cm^2^V^−1^s^−1^), the μ_e_ values of the corresponding BHJ composites are very different due to the different nanoscale morphologies. For example, the μ_e_ of PTB7-Th:PyF5 (1:1) and PTB7-Th:FAP1 (1:1) are in the order of 10^−7^–10^−6^ cm^2^V^−1^s^−1^ and these are in sharp contrast with the much higher μ_e_ of PTB7-Th:PC_61_BM which is ~10^−4^ cm^2^V^−1^s^−1^. As the miscibility of PTB7-Th is higher with PyF5 and FAP1 than with PC_61_BM, PTB7-Th:PyF5 and PTB7-Th:FAP1 blends require significantly higher fullerene loadings to reach comparably high electron mobilities as for PTB7-Th:PC_61_BM blends. The authors, therefore, concluded that BHJ composites with good polymer–fullerene miscibility require higher fullerene loadings than composites with a tendency to phase separate.

Nagarjuna et al. [103] synthesized the fullerene DIF-ful-C_60_ (Figure 5) and tested it with PTB7, as previously mentioned and with PTB7-Th. The optimized PTB7-Th: DIF-ful-C_60_ OPV devices showed a highest PCE of 8.6% with a *J_sc_* of 16.01 mA·cm^−2^, *V_oc_* of 0.82 V and a high *FF* of 65.5%, whereas the reference solar cell made from PTB7-Th:PC_61_BM blends showed a highest efficiency of 7.9%, with reduced *J_sc_* of 15.14 mA·cm^−2^ and *FF* of 62.1%. In a different work, the same authors [119] synthesized the fullerene derivative CN-PC_71_BM and optimized its application in PTB7-Th-based devices with an inverted structure. The best devices displayed a PCE of 8.2% and were obtained using a D:A mass ratio of 1:1.5, dissolved in CB and containing 3 vol % of CLN as additive.

## 6. Devices Based on PffBT4T-2OD

The small band gap donor polymer poly[(5,6-difluoro-2,1,3-benzothiadiazol-4,7-diyl)-alt-(3,3‴-di(2-octyldodecyl)2,2′;5′,2″;5″,2‴-quaterthiophen-5,5‴-diyl)] (PffBT4T-2OD) has recently attracted attention due to its potential to fabricate high performing OPV devices. PffBT4T-2OD, also known as PCE11, exhibits relatively high SCLC hole mobility of 1.5–3.0 × 10^−2^ cm^2^V^−1^s^−1^ [120] due to its high crystallinity. These properties, together with its tendency to form relatively pure polymer domains when blended with fullerenes, allow it to perform well in an OPV device, when used in relatively thick BHJ layers (~300 nm) with higher light absorption capabilities. PffBT4T-2OD when in solution also exhibits a peculiarly strong temperature dependent aggregation behaviour, forming a gel at room temperature. Consequently, PffBT4T-2OD based devices need to be always cast from warm solutions (>60 °C), which then aggregate or crystallize during cooling and film forming processes.

Table 3, at the end of this chapter, summarizes the most relevant and recent device efficiency results obtained with OPVs based on PffBT4T-2OD:fullerene BHJs.

### 6.1. PffBT4T-2OD Devices with PC_61_BM and PC_71_BM

Ma et al. [121] investigated the influence of several processing parameters (solution temperature, concentration, spin-rate and solvent quality), as well as the influence of polymer molecular weight on the morphology and performance of PffBT4T-2OD:PC_71_BM devices. Here, it was found that the molecular orientation and molecular packing can be tuned by adjusting the solution temperature and the spin rate during spin-coating of the BHJ, with a low solution temperature and low spinning rate inducing highly ordered face-on polymer packing and a high solution temperature and a high spinning rate producing poorly ordered edge-on polymer packing. The best device performance (average PCE of 10.3%) was achieved using films spun-cast at 800 rpm from a solution at 100 °C, creating a smooth film containing sufficient aggregates to yield a favourable morphology.

Zhang et al. [122] studied the impact, on the morphology and efficiency of PffBT4T-2OD:PC_71_BM devices, of depositing the BHJ from different co-solvent mixtures namely: (i) o-xylene with 1 vol % p-anisaldehyde; (ii) CB with 3 vol % DIO; (iii) o-DCB with 3 vol % DIO and (iv) a mixture of 48.5 vol % CB and 48.5 vol % o-DCB with 3 vol % DIO. The best device efficiencies (average PCE = 9.07%) were obtained using the mixture CB:o-DCB:DIO. The authors attributed this higher efficiency to the higher crystallinity of PffBT4T-2OD and higher absorption density of the BHJ when processed with the ternary solvent combination.

The impact of four different additives (ODT, DIO, diphenylether (DPE) and CLN), added to the photovoltaic ink solution in 3 vol %, on the morphology and performance of the resultant PffBT4T-2OD:PC_71_BM devices, was studied by Zhao et al. [123]. The best devices displayed a PCE of 10.23% and were obtained using CN as additive. GIWAXS data showed that films processed with CN additive possess enhanced crystallinity of PffBT4T-2OD in the (100) direction corresponding to the alkyl stacking peak located at a q value of 0.29 Å^−1^, which facilitates charge transport within the BHJ.

Zhang et al. [39] helped elucidating the mechanism of action of additives studying the effect of using 3 vol % of DIO in the preparation of PffBT4T-2OD:PC_71_BM devices. The results showed that PCE can increase by ~20%, from 7.2% to above 8.95%, as a result of the coarsening of the phase domains induced in the film by DIO and thermal annealing, through a mechanism of transient plasticisation. Furthermore, the authors showed that DIO can be removed from the film by a thermal annealing process at temperatures below 100 °C and that there is an interplay between the evaporation rate of DIO and the rate of domain coarsening in the plasticized film.

**Figure 9:**
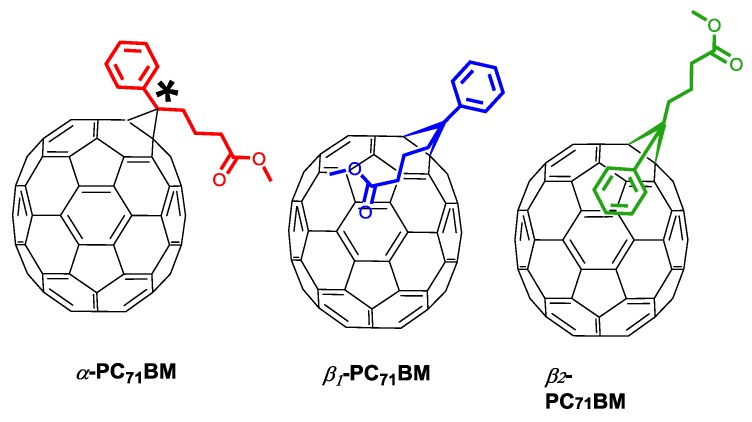
Different isomers of PC_71_BM. Adapted from reference [124].

The effect of different isomers of PC_71_BM on the photovoltaic properties of PffBT4T-2OD:PC_71_BM blend films was investigated by Umeyama et al. [124]. It should be noted here that the PC_71_BM normally used in OPV devices consists of an isomer mixture of *α*-type isomers (80–90%) and *β*-type isomers (10–20%). The *α*-isomer is composed by two enantiomers, while the *β*-isomer consists of two diastereomers in which the phenyl group is extended toward opposite directions (Figure 9) [125]. Interestingly, the authors found that one of the diastereomers of the *β*-isomer (isomer *β*_1_) shows an extraordinary cohesion in the blended film, deteriorating the OPV device performance. In fact, the PffBT4T-2OD devices based on *β*_1_-PC71BM exhibited an extremely low PCE of 0.43%. By contrast, OPV devices using the remaining pure isomers exhibited much higher PCE, namely *α*-PC_71_BM (8.80%), *β*_2_-PC_71_BM (8.75%) and these slightly surpass the device with using the normal PC_71_BM isomer mixture (8.46%) [124]. The authors concluded that decreasing the amount of a diastereoisomer of *β*_1_-PC_71_BM with high aggregation tendency improves the photovoltaic performances [124].

Recently Li et al. [126] reported high efficiency ternary polymer solar cells, based on the system PffBT4T-2OD:PC_71_BM and containing the amorphous medium band gap conjugated polymer PCDTBT8 as a third minority component. Different mass fractions of PCDTBT8 were tested (0, 5, 10, 15, 20 and 30%) and the most efficient devices (average PCE of 11.0%) were prepared using 15 wt % of PCDTBT8. Morphological investigation showed that the third component PCDTBT8 locates at the interface between PffBT4T-2OD and PC_71_BM, reduces the fullerene aggregation networks leading to increased exciton dissociation and additionally does not disrupt the crystallization of PffBT4T-2OD maintaining a good hole mobility (without PCDTBT8: μ_h_ = 0.0113 cm^2^V^−1^s^−1^; with 15 wt % PCDTBT8: μ_h_ = 0.0092 cm^2^V^−1^s^−1^).

### 6.2. PffBT4T-2OD Devices with Other Fullerenes

In one of the most extensive polymer:fullerene combination studies ever reported in the organic photovoltaics literature, Liu et al. [120] have tested the use of a large number of different fullerene acceptors (PC_71_BM, PC_61_BM, TC_71_BM, PC_61_PM, TC_61_PM and ICMA—Figure 5) in devices based on the polymer PffBT4T-2OD and on other family related polymers. Their results have shown that the efficiency of the devices obtained using different fullerenes were all very similar (average values within the range 9.3–10.3%) and this observation led these authors to conclude that the aggregation behaviour of PffBT4T-2OD is insensitive to the presence of the fullerene acceptor and can be efficiently used to control the morphology of the corresponding BHJs. This has permitted a near-ideal polymer:fullerene morphology to be created (containing highly crystalline, preferentially orientated, yet small polymer domains) by control over polymer aggregation during solution casting. The highest efficiency devices with an average PCE of 10.3% were obtained with the fullerene TC_71_BM (Figure 5).

More recently Zhang et al. [127] studied the effect of three different fullerene acceptors (PC_71_BM, PC_61_BM and ICBA) on the performance of solar cells based on PffBT4T-2OD. Here it should be noted that the fullerene bisadduct ICBA has not been included in the previous extensive work by Liu et al. [120]. These authors have found that although PffBT4T-2OD:ICBA devices had a much higher *V_oc_* (0.94 V) than the corresponding devices using PC_71_BM (0.76 V) and PC_61_BM (0.77 V), their efficiency (average PCE of 2.78%) was much lower than the efficiency of corresponding devices based on PC_71_BM (average PCE of 8.93%) and PC_61_BM (average PCE of 8.15%). Interestingly, these results do not seem to support the conclusions of Liu et al. Morphological characterization has shown that although the size of phase domains is very similar in the three different BHJs, the fullerene aggregates in the ICBA-based films have a reduced degree of order. Furthermore, due to the high LUMO level of ICBA, the corresponding BHJs are characterised by a lower initial exciton dissociation and this associated with the reduced ordering within the ICBA domains results in increased geminate recombination of the photogenerated electrons in the fullerene-rich domains and a consequently reduced PCE of the corresponding devices.

## 7. Conclusions and Perspectives

Although non-fullerene acceptors have now surpassed fullerenes in terms of performance in OPVs, the research for developing new fullerenes and the morphological and efficiency optimization of polymer:fullerene solar cells, remains very intense. This article reviews the most relevant OPV studies, from a morphological viewpoint, involving BHJs of conjugated polymers PTB7, PTB7-Th and PffBT4T-2OD with fullerene acceptors, over the last 3 years (from 2016 onwards).

Considering the efficiency improvements over the last three years, these were predominately driven by the optimization of the morphology at the nanoscale level of the photoactive blend. Some of the most relevant achievements reviewed in this article include the development of single-junction OPVs with PCE > 14% based on ternary BHJs and also new developments on understanding the mechanism of the additives as morphological enhancers.

Although many new different fullerenes have been synthesized and tested for OPV devices, in general these did not outperform the standard PC_71_BM, which keeps unthreatened its leading position, as the most reliable and popular fullerene within the OPV research. The addition of two or more addends to the fullerene buckyball is known to increase its LUMO level and consequently increase the *V_oc_* of the corresponding OPV devices. However, most devices based on these multi-adduct fullerenes fail to maintain high *J_sc_* and *FF* values and as a result the overall device efficiency is usually worse than in reference devices with the standard PC_71_BM. The addition of two or more adducts usually degrades the device efficiency for two main reasons: they decrease the overall crystallinity of the fullerene and as a result the electron mobility decreases; and, second, they affect drastically the degree of phase separation from the polymer and the consequent polymer:fullerene morphology at the nanoscale. The major challenge in this topic lies in finding the optimal compromise among a combination of fullerene properties that often works in opposite directions.

As reported in this review, the strategies followed, so far, for synthesizing new fullerene acceptors for OPVs have been largely based on single-molecule properties and nowadays theoretical calculations (*ab initio*) can, for example, estimate with high accuracy the HOMO-LUMO levels of new fullerene acceptors. However, single-molecule properties cannot account for the whole story, as they completely neglect all aspects related to molecular interactions and structural organization in the solid state, including degree of crystallinity and crystallographic structure, which are crucially important for properties such as the electron mobility. Therefore, one research direction that is still clearly underexplored and poorly understood concerns the fundamental study of the relationship between the chemical structure of single fullerene molecules, their 3-D molecular packing in the solid state and the corresponding electron mobility. This will require the combination of a battery of experimental and theoretical techniques including: organic synthesis of new fullerenes; X-ray diffraction characterization of solid state structures; computational modelling (*ab initio*, molecular dynamics); and measurement of charge mobilities, among others. This fundamental knowledge will certainly prove very useful in designing better strategies for the synthesis of new fullerenes for highly efficient OPV applications.

Recent research trends suggest that the future of fullerenes in the OPV field might be not on their use as main electron acceptors but rather in ternary BHJs (polymer:acceptor1:acceptor 2) in combination with other non-fullerene small molecule acceptors.

Most optimized morphologies still cannot remain stable for long periods of time due to molecular diffusion and molecular degradation of the BHJ components under real device operation. Therefore, further research is needed for improving the morphological stability of these BHJs. Additionally, the technology needs to be scaled up into much larger areas using roll-to-roll processing. Namely, further research is still needed for replacing the typical halogenated solvents by other more environmentally friendly solvents.

The development and commercialization of next-generation OPVs is a highly multidisciplinary research subject and only through a sustained cooperative effort involving the expertise of material chemists, physicists and engineers it will be possible to succeed.

## Figures and Tables

**Table 1:** Summary of the most relevant device figures of merit and efficiency results obtained with PTB7:fullerene BHJs.

Acceptor	D:A Wt.	Solvent	Additive	J_SC_ (mA cm^−2^)	V_OC_ (V)	FF (%)	PCE (%) Best (average)	Device Structure	Device Area (mm^2^)	Obs.	Ref
PC_71_BM	1:1.5	CB (100 vol %)	-------	10.20	0.76	50.52	3.92	Standard ITO/PEDOT:PSS/BHJ/Ca/Al	10.0	----	[85]
		CB (97 vol %)	DIO (3 vol %)	14.50	0.74	68.97	7.40				
PC_71_BM	1:1.5	CB (97 vol %)	DIO (3 vol %)	15.46	0.76	68 ± 1	7.94	Standard ITO/PEDOT:PSS/BHJ/Ca/Al	4.5	(a)	[86]
PC_71_BM	1:1.5	o-DCB (100 vol %)	-------	18.51	0.76	60	8.50	Standard ITO/PEDOT:PSS/BHJ/Ca/Al	4.5	(b)	[71]
PC_71_BM	1:1.5	CB (97 vol %)	DIO (3 vol %)	15.4	0.759	70.6	8.24	Standard ITO/PEDOT:PSS/BHJ/PFN/Ca/Al	16	(c)	[87]
				17.2	0.740	72.0	9.15	Inverted ITO/PFN/BHJ/MoO_3_/Al			
PC_71_BM	1:1.5	CB (100 vol %)	-------	11.43	0.70	46	3.99 (3.68)	Standard ITO/PEDOT:PSS/BHJ/Ca/Al	7.5	(d)	[61]
		CB (97 vol %)	SH-na (3 vol %)	15.67	0.79	70	8.75 (8.42)	Standard ITO/PEDOT:PSS/BHJ/Ca/Al			
PC_71_BM	1:1.5	CB (95 vol %)	DPE (2 vol %)+ DIO (3 vol %)	18.1	0.72	71.0	9.55 (9.25)	Inverted ITO/ZnO/BHJ/MoO3/Ag	-----	----	[91]
PC_71_BM	1:1.5	CB 97 vol %	DIO 3 vol %	17.49	0.764	66.1	8.84	Inverted ITO/PEIE/BHJ/MoO3/Ag	9	----	[92]
PC_71_BM	1:1.5	CB (91 vol %)	FA (6 vol %) + DIO (3 vol %)	24.11	0.72	52.11	9.04	Standard ITO/PEDOT:PSS/BHJ/Li/Al	4	(e)	[93]
PC_71_BM	1:1.5	CB (97 vol %)	DIO (3 vol %)	15.2	0.73	67.8	7.53 (7.40)	Standard ITO/PEDOT:PSS/BHJ/PFN/Al	16	----	[94]
		CB (95 vol %)	CBA (5 vol %)	16.7	0.75	73.0	9.11 (8.99)	Standard ITO/PEDOT:PSS/BHJ/PFN/Al			
PC_71_BM	1:1.5	o-DCB (97 vol %)	DIO (3 vol %)	14.2	0.74	60.0	6.30	Standard ITO/PEDOT:PSS/BHJ/Ca/Al	10	----	[98]
ICBM				15.4	0.79	55.0	6.67				
Fullerene 4	1:1.5	CB (97 vol %)	DIO (3 vol %)	15.48	0.749	63.3	7.34	Inverted ITO/PFN/BHJ/MoOx/Al	9	----	[99]
Fullerene 5				14.21	0.760	67.3	7.27				
Fullerene 6				14.03	0.797	61.0	6.83				
PC_61_BM				14.29	0.740	66.5	7.03				
IC_60_MA-2C	-----	CB (97 vol %)	DIO (3 vol %)	14.2	0.77	55	6.0	Inverted ITO/ZnO/BHJ/MoO3/Al	7	----	[100]
IC_60_MA-3C				12.9	0.79	50	5.1				
IC_60_MA-4C				13.7	0.77	61	6.5				
PC_61_BM				14.6	0.76	62	6.8				
Fullerene 1e	1:1.5	CB 95 vol %	DIO 5 vol %	12.3	0.825	53.3	5.4	Standard ITO/PEDOT:PSS/BHJ/Ca/Al	7.2	----	[101]
PC_61_BM				12.1	0.760	64.4	5.9				
Fullerene N3	1:1.5	o-DCB 97 vol %	DIO 3 vol %	9.73	0.812	52.1	4.12	Inverted ITO/ZnO/BHJ/MoO_3_/Ag	-----	----	[102]
Fullerene N6				9.06	0.805	50.2	3.64				
DIF-ful-C_60_	1:1.5	o-DCB 97 vol %	DIO 3 vol %	15.97	0.82	51	6.8 (6.5)	Inverted ITO/ZnO/BHJ/MoO_3_/Ag	-----	(f)	[103]
PC_61_BM				15.40	0.70	58	6.2 (5.9)				
PC_71_BM	1:1.5	CB (97 vol %)	DIO (3 vol %)	14.99	0.701	68.8	7.35 (7.23)	Standard ITO/PEDOT:PSS/BHJ/Ca/Al	4	----	[105]
PC_71_BM(85%) + ICBA (15%)				16.32	0.720	69.2	8.24 (8.13)				
PC_71_BM	1:1.5	o-DCB (97 vol %)	DIO (3 vol %)	17.4	0.76	64.8	8.57	Inverted ITO/ZnO/BHJ/MoO_3_/Ag	9	----	[106]
PC_71_BM (85%) + NCBA (15%)				18.6	0.78	67.9	9.85				

Observations: (a) Methanol treatment; (b) PTB7 high M_W_ of 128 kg/mol; (c) PFN is used as surface modifier; (d) With solution dipping; (e) FA is Formic acid; (f) For PTB7:DIF-ful-C60 based OPVs, the D:A mass ratio was optimized and 1:1.5 showed the best performances.

**Table 2:** Summary of the most relevant device figures of merit and efficiency results obtained with PTB7-Th:fullerene BHJs.

Acceptor	D:A (*w*/*w*)	Solvent	Additive	J_SC_ (mA cm^−2^)	V_OC_ (V)	FF (%)	PCE (%) Best (average)	Device Structure	Device Area (mm^2^)	Obs.	Ref.
PC_71_BM	1:1.5	o-DCB (99 vol %)	DIO (1 vol %)	16.03	0.786	65.12	8.21	Standard ITO/PEDOT:PSS/BHJ/Ca/Al	4	----	[108]
		o-DCB (97 vol %)	DIO (3 vol %)	16.86	0.784	68.16	9.00				
		o-DCB (95 vol %)	DIO (5 vol %)	14.59	0.779	64.73	7.35				
PC_71_BM	1:1.5	CB (100 vol %)	---------	16.2	0.80	49	6.4 (6.1)	Inverted ITO/PEIE/BHJ/MoO_3_/Ag	4.5	----	[109]
		CB (97 vol %)	DIO (3 vol %)	18.1	0.79	66	9.5 (9.3)				
PC_71_BM	1:1.5	o-DCB (100 vol %)	---------	17.0	0.83	58.1	8.2 (8.1)	Standard ITO/PEDOT:PSS/BHJ/C_60_-N/Al	4	(a)	[110]
		o-DCB (97 vol %)	NMP (3 vol %)	18.0	0.82	62.1	9.2 (8.9)				
		o-DCB (97 vol %)	DIO (3 vol %)	17.9	0.82	64.5	9.5 (9.2)				
		o-DCB (97 vol %)	DIO 1.5 vol % + NMP 1.5 vol %	19.1	0.82	69.1	10.8 (10.4)				
PC_71_BM	1:1.5	CB (97 vol %)	DIO (3 vol %)	15.97	0.79	60.12	7.58 (7.51)	ITO/PEDOT:PSS/BHJ/BCP/Ag	12.56	(b)	[111]
				17.74	0.784	64.11	8.95 (8.89)				
PC_71_BM	1:0.5	o-DCB (100 vol %)	---------	6.69	0.78	31	1.60	Standard ITO/PEDOT:PSS/BHJ/Ca/Al	13	(c)	[62]
	1:1			12.59	0.80	50	5.04				
	1:1.5			19.01	0.80	53	8.08				
	1:2			18.15	0.79	65	9.38				
	1:3			10.41	0.77	43	3.49				
PC_71_BM (0.45 wt) + CO_i_8DFIC (1.05 wt)	1:1.5	CB (99 vol %)	DIO (1 vol %)	28.20	0.70	71.0	14.08	Inverted ITO/ZnO/BHJ/MoO_3_/Ag	4	----	[7]
PC_71_BM	1:1.5	CB (97 vol %)	DIO (3 vol %)	16.6	0.77	71.3	9.1 (8.8)	Inverted ITO/ZnO/BHJ/MoO_3_/Ag	8	(d)	[112]
PC_71_BM	1:1.5	o-DCB (97 vol %)	DIO (3 vol %)	16.55	0.80	71	9.68 (9.42)	Inverted ITO/ZnO/BHJ/MoO_3_/Ag	----	(e)	[59]
	1:3			14.53	0.80	73	8.73 (8.51)				
PC_71_BM	1:1.8	CB (97 vol %)	DIO (3 vol %)	----	----	----	9.25	Standard ITO/PEDOT:PSS/BHJ/LiF/Al	8	(f)	[113]
				----	----	----	10.4	Inverted ITO/ZnO/BHJ/MoO_3_/Ag			
PC_71_BM	1:1.5	CB (100 vol %)	---------	9.7	0.83	47.5	3.8 (3.3)	Inverted ITO/PEIE/BHJ/MoO_3_/Ag	----	----	[114]
		CB (97 vol %)	DIO (3 vol %)	16.1	0.79	64.8	8.3 (8.1)				
PC_71_BM	1:1.5	CB (97 vol %)	DIO (3 vol %)					Standard ITO/PEDOT:PSS/BHJ/Ca/Ag	4.5	----	[115]
								Inverted ITO/TiO_2_/BHJ/MoO_x_/Ag			
PC_71_BM	1:1.5	CB (97 vol %)	DIO (3 vol %)	16.85	0.801	70.27	9.72 (9.49)	Inverted ITO/ZnO/BHJ/MoO_3_/Ag	6	----	[116]
	1:1.5	1,2-DCB (100 vol %)	-----------	14.93	0.802	65.28	7.82	Inverted ITO/PFN/BHJ/MoO_3_/Ag			
	1:2.0			16.01	0.802	72.28	9.59 (9.28)				
PC_61_BM	1:1.5	CB (97 vol %)	DIO (3 vol %)	14.6	0.81	64	(7.6)	Inverted ITO/PEIE/BHJ/MoO_3_/Ag	10	----	[117]
PC_71_BM				17.7	0.80	66	(9.4)				
ICBA				13.3	1.0	53	(7.1)				
PC_61_BM	1:1.5	CB (97 vol %)	DIO (3 vol %)	14.0	0.80	65	7.3	Inverted ITO/ZnO/BHJ/MoO_x_/Ag	10.4	(g)	[118]
PyF5	1:2			13.7	0.84	56	6.5				
FAP1	1:2			12.7	0.87	55	6.1				
PC_61_BM	1:1.5	o-DCB (97 vol %)	DIO (3 vol %)	15.14	0.84	62	7.9 (7.7)	Inverted ITO/ZnO/BHJ/MoO_3_/Ag	----	----	[103]
DIF-ful-C_60_				16.01	0.82	65	8.6 (8.4)				
PC_71_BM	1:1.5	CB (97 vol %)	CLN (3 vol %)	14.0	0.795	48.8	5.4	Inverted ITO/ZnO/BHJ/MoO_3_/Ag	10	----	[119]
CN-PC_71_BM				13.5	0.898	68.0	8.2				

Observations: (a) NMP is N-methyl pyrrolidine. (b) Reference device (lower PCE) without 1-bromo-4-nitrobenzene; device with higher PCE contains 1-bromo-4-nitrobenzene (15 wt% active layer). (c) The BHJ was surface washed with ethanol before top electrode deposition. (d) Devices subjected to different annealing temperatures were tested (RT, 70, 100, 120 and 150 °C). Best devices were obtained at RT. Only the performance of these is shown in this table. (e) For devices with D:A mass ratio 1:1.5, the following different BHJ thickness were tested: 70, 90, 120, 180 and 270 nm. Best devices were obtained with an optimized BHJ thickness of 90 nm. Only the performance of these is shown in this table. For devices with D:A ratio 1:3, the following different BHJ thickness were tested: 120, 180 and 270 nm. Best devices were obtained with an optimized BHJ thickness of 120 nm. Only the performance of these is shown in this table. (f) The performance data shown here were obtained with an optimized BHJ thickness of 116 nm in the standard devices and of 76 nm in the inverted devices. (g) The PTB7-Th:fullerene ratios were optimized and only devices with the highest efficiency ratios are shown: 1:1.5 for PC_61_BM and 1:2 for PyF5 and FAP1.

**Table 3:** Summary of the most relevant device figures of merit and efficiency results obtained with PffBT4T-2OD:fullerene BHJs.

Acceptor	D:A (*w*/*w*)	Solvent	Additive	*J_SC_* (mA cm^−2^)	*V_OC_* (V)	*FF* (%)	*PCE* (%) Best (Average)	Device Structure	Device Area (mm^2^)	Obs.	Ref.
PC_71_BM	1:1.2	CB (48.5 vol %) + DCB (48.5 vol %)	DIO (3 vol %)	18.5	0.79	71	10.3	Inverted ITO/ZnO/BHJ/MoO_3_/Al	5.64	(a)	[121]
PC_71_BM	1:1.2	CB (48.5 vol %) + DCB (48.5 vol %)	DIO (3 vol %)	18.19	0.76	66.6	9.16 (9.07)	Inverted ITO/ZnO/BHJ/MoO_3_/Ag	2	(b)	[122]
PC_71_BM	1:1.3	CB (48.5 vol %) + DCB (48.5 vol %)	CLN (3 vol %)	17.75	0.79	73.1	10.23 best	Standard ITO/PEDOT:PSS/BHJ/LiF/Al	4	(c)	[123]
PC_71_BM	1:1.2	CB (50 vol %) + DCB (50 vol %)	-----------	16.13	0.72	62.93	7.29 (7.08)	Standard ITO/PEDOT:PSS/BHJ/Ca/Al	2.6	(d)	[39]
		CB (48.5 vol %) + DCB (48.5 vol %)	DIO (3 vol %)	17.33	0.75	68.34	8.90 (8.73)				
PC_71_BM	------	CB (48.5 vol %) + DCB (48.5 vol %)	DIO (3 vol %)	19.5	0.75	72.2	10.57 (10.3)	Inverted ITO/TiO_2_/BHJ/MoO_3_/Ag	-----	(e)	[126]
				18.8	0.79	74.7	11.17 (11.0)				
TC_71_BM	1:1.2	CB (48.5 vol %) + DCB (48.5 vol %)	DIO (3 vol %)	18.8	0.77	75	10.8 (10.3)	Inverted ITO/ZnO/BHJ/MoO_3_/Al	5.9	-----	[120]
PC_71_BM				18.4	0.77	74	10.5 (10.2)				
PC_61_PM				17.7	0.77	76	10.4 (10.1)				
ICMA				16.4	0.78	77	9.8 (9.4)				
TC_61_PM				17.4	0.75	74	9.7 (9.3)				
PC_61_BM				17.1	0.77	73	9.6 (9.3)				
PC_71_BM	1:1.2	CB (48.5 vol %) + DCB (48.5 vol %)	DIO (3 vol %)	17.3	0.76	70	9.31 (8.93)	Standard ITO/PEDOT:PSS/BHJ/Ca/Al	2.6	-----	[127]
PC_61_BM				16.3	0.77	67	8.46 (8.15)				
ICBA				7.5	0.94	45	3.19 (2.78)				

Observations: (a) Different processing conditions (spin-rate, solution temperature) were tested. Only the devices with the best performance are shown and these had a thickness of 300 nm and were obtained with a solution temperature of 100 °C and a spin rate of 800 rpm. (b) Different solvent combinations were tested. Only the devices with the best performance are shown and these were obtained using a mixture of CB:DCB (1:1 *v*/*v*) with 3 vol % DIO. (c) Different additives were tested. Only the device data with the best performance are shown and these were obtained using 1-chloro-naphthalene as additive. (d) Reference device without DIO was not annealed. Device with DIO was annealed for 5 min at 100 °C. (e) Reference device without PCDTBT8 shown on top and device with 15 wt% PCDTBT8 on bottom.
